# A Hybrid In Silico Approach for Identifying Dual VEGFR/RAS Inhibitors as Potential Anticancer and Anti-Angiogenic Agents

**DOI:** 10.3390/ph18101579

**Published:** 2025-10-18

**Authors:** Alessia Bono, Gabriele La Monica, Federica Alamia, Dennis Tocco, Antonino Lauria, Annamaria Martorana

**Affiliations:** 1 Dipartimento di Scienze e Tecnologie Biologiche Chimiche e Farmaceutiche “STEBICEF”, University of Palermo, Viale delle Scienze, Ed. 17, 90128 Palermo, Italy; alessia.bono01@unipa.it (A.B.); gabriele.lamonica01@unipa.it (G.L.M.); federica.alamia01@unipa.it (F.A.); dennis.tocco@you.unipa.it (D.T.); annamaria.martorana@unipa.it (A.M.); 2 Fondazione Umberto Veronesi (FUV), Via Solferino 19, 20121 Milano, Italy; 3 National Biodiversity Future Center (NBFC), Piazza Marina 61, 90133 Palermo, Italy

**Keywords:** angiogenesis, VEGFR-2, K-RAS, anticancer activity, inhibitors, NCI database

## Abstract

**Background**: Angiogenesis, the physiological process by which new blood vessels originate from pre-existing ones, can be triggered by tumor cells to promote the growth, survival, and progression of cancer. Malignant tumors require a constant blood supply to meet their needs for oxygen and nutrients, making angiogenesis a key process in tumor development. Its pathologic role is caused by the dysregulation of signaling pathways, particularly those involving VEGFR-2, a key mediator of angiogenesis, and the K-RAS G12C mutant, a promoter of VEGF expression. Given their critical involvement in tumor progression, these targets represent promising candidates for new cancer therapies. **Methods and Results**: In this study, we applied an in silico hybrid and hierarchical virtual screening approach to identify potential dual VEGFR-2/K-RAS G12C inhibitors with anticancer and antiangiogenic properties. To this end, we screened the National Cancer Institute (NCI) database through ADME filtering tools. The refined dataset was then submitted to the ligand-based Biotarget Predictor Tool (BPT) in a multitarget mode. Subsequently, structure-based analysis, including molecular docking studies on VEGFR and K-RAS G12C, was performed to investigate the interactions of the most promising small molecules with both targets. **Conclusions**: Finally, the molecular dynamics simulations suggested compound **737734** as a promising small molecule with high stability in complex with both VEGFR-2 and K-RAS G12C, highlighting its potential as a dual-target inhibitor for cancer therapy.

## 1. Introduction

Cancer remains one of the leading causes of morbidity and mortality worldwide. According to the most recent GLOBOCAN 2022 data (Update of the GLOBOCAN 2022 database version 1.1.), approximately 19.96 million new cancer cases and 9.74 million cancer-related deaths were reported globally, with incidence and mortality rates continuing to rise in many regions [1]. In the European Union alone, an estimated 2.7 million new cases and 1.3 million deaths were recorded in 2022, confirming cancer as a major public health concern (https://ecis.jrc.ec.europa.eu/, accessed on 11 October 2025).

Among the biological mechanisms that sustain tumor growth and progression, angiogenesis plays a pivotal role and is closely associated with poor prognosis and increased mortality in several malignancies, including lung, colorectal, and breast cancers [2,3,4,5].

Angiogenesis is the physiological process through which new blood vessels arise from pre-existing vasculature, following vasculogenesis, the de novo formation of blood vessels, to build up the vascular network during embryonic development. This complex phenomenon is also essential for tissue growth and wound healing [6].

However, angiogenesis also plays a role in pathological processes. In contrast to its physiological counterpart, pathological angiogenesis is dysregulated and actively contributes to the growth and progression of malignant tumor cells. The process of angiogenesis induced by tumors begins when the cancer mass increases in size, and thus the need for oxygen and nutrients increases, thereby requiring the growth of new blood vessels. This triggers a phenomenon known as the angiogenic switch, which occurs following sequential steps, leading to a disruption in the balance between pro- and anti-angiogenic factors that regulate the process [7,8]. In particular, the process begins with the onset of hypoxia within the tumor microenvironment, which promotes the expression of Hypoxia-Inducible Factors (HIFs), such as *HIF-1α*. These, in turn, act as a transcription factor and induce the secretion of Vascular Endothelial Growth Factors (VEGFs), such as *VEGF-A*, and Fibroblast Growth Factors (FGFs) from the surrounding tissue. Subsequently, endothelial cells activated by these pro-angiogenic signals undergo proliferation and stabilization. The persistent overexpression of VEGFs and FGFs continues to drive the angiogenic process, leading to the formation of tumor-associated blood vessels that are structurally abnormal, characterized by disorganization, malformation, tortuosity, fragility, and frequent absence of pericytes [9,10].

The principal signaling pathway regulating angiogenesis is mediated by the VEGF family, which includes the glycoproteins VEGF-A to VEGF-D and the Placental Growth Factor (PlGF). As previously mentioned, the dominant inducer of angiogenesis is VEGF-A, which exerts its effects through direct binding to VEGFRs, Receptor Tyrosine Kinases (RTKs), specifically VEGFR-1, VEGFR-2, and VEGFR-3, located on the cellular membrane of endothelial cells. Notably, although VEGF-A targets both VEGFR-1 and VEGFR-2, VEGFR-1 exhibits lower kinase activity, characterized by weak ligand-dependent tyrosine kinase autophosphorylation, and often acts as a decoy receptor, sequestering VEGF-A and preventing its interaction with VEGFR-2. Therefore, VEGFR-2 emerges as the principal signaling receptor responsible for mediating vascular endothelial cell mitogenesis and increasing vascular permeability [11,12]. Activation of the VEGFR-2 pathway involves the binding of its endogenous ligand, leading the protein to undergo homodimerization or heterodimerization, predominantly with VEGFR-3, and triggering autophosphorylation of specific intracellular tyrosine residues, with five major sites identified: Tyr^951^, Tyr^1054^, Tyr^1059^, Tyr^1175^, and Tyr^1214^.

The downstream signaling is initiated by the recruitment of various intracellular proteins to the phosphorylated tyrosine residues, a process mediated by their SH2 domains. This results in the activation of multiple signaling pathways, including the PI3K-AKT-mTOR pathway, essential for cell survival; the Cdc42-p38-MAPK-HSP27 pathway, which plays a central role in the cell migration process; and the RAS-RAF-MEK-ERK1/2 cascade, primarily responsible for the proliferation component during angiogenesis [13,14,15,16].

Among the proteins involved in these pathways, K-RAS critically influences the formation of new vessels from the pre-existing vasculature by upregulating the expression of VEGF. Wild-type K-RAS is a small GTPase that cycles between active (GTP-bound) and inactive (GDP-bound) states, regulating essential cellular processes such as proliferation, differentiation, and survival under physiological conditions [17,18].

However, mutations in *KRAS*, particularly the G12C substitution, lead to a constitutively active form of the protein that remains locked in its GTP-bound state, thereby sustaining uncontrolled RAS/MAPK signaling. This aberrant activation promotes tumor growth, angiogenesis, and therapeutic resistance. Due to its critical oncogenic role, K-RAS G12C has emerged as one of the most relevant molecular targets in precision oncology [19,20].

This biological distinction is crucial in the context of anti-angiogenic drug design, as the G12C mutant not only amplifies VEGF expression but can also enhance VEGFR-2 activation, resulting in a self-reinforcing loop of angiogenesis and proliferation [21,22]. Therefore, simultaneous targeting of VEGFR-2 and mutant K-RAS represents a promising strategy to disrupt the interlinked signaling networks driving tumor progression [23,24,25,26,27].

**Figure 1:**
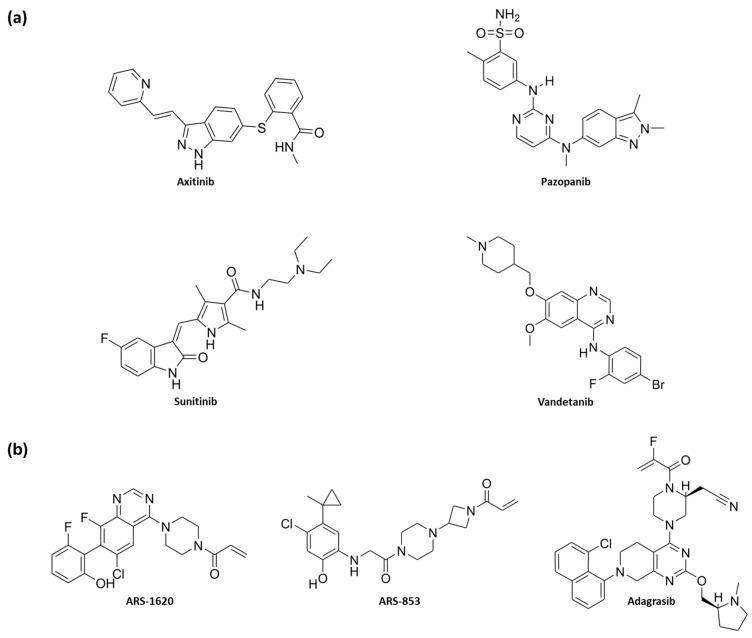
(**a**) 2D chemical structures of compounds **axitinib**, **pazopanib**, **sunitinib**, and **vandetanib**, approved as VEGFR-2 inhibitors; (**b**) 2D chemical structures of compounds **ARS-1620**, **ARS-853**, and **adagrasib**, K-RAS inhibitors (among these, only **adagrasib** is approved).

Inhibitors targeting VEGFR-2 are classified into three categories: type I, type II, and type III. Type I inhibitors act by blocking the active conformation of the ATP-binding pocket; type II inhibitors, in contrast, inhibit the inactive conformation of the kinase; and type III inhibitors are covalent inhibitors that bind the kinase irreversibly. Type I inhibitors are the most numerous among those approved, including **axitinib**, **pazopanib**, **sunitinib**, and **vandetanib** (Figure 1a). However, despite their clinical use, these agents have shown limited long-term efficacy, often due to the development of resistance and mechanism-related toxicities, such as hypertension, fatigue, and cardiotoxicity [28,29,30]. Resistance mechanisms typically involve reactivation of downstream RAS/RAF/MEK/ERK signaling or the upregulation of compensatory pro-angiogenic pathways, ultimately restoring tumor vascularization and proliferation.

In this context, a dual-targeting strategy simultaneously inhibiting VEGFR-2 and K-RAS may offer synergistic therapeutic benefits, as it could suppress both angiogenesis and RAS-driven tumor cell proliferation. Such a combined inhibition could therefore enhance anticancer efficacy and potentially overcome resistance to anti-angiogenic monotherapies.

K-RAS, traditionally considered “undruggable” [31] due to its GTP binding site exhibiting high specificity in the picomolar range, has more recently been targeted through the identification of the switch II pocket as a receptive non-covalent binding site, with ligands such as **ARS-1620**, **ARS-853**, and **adagrasib** (Figure 1b). Nonetheless, K-RAS inhibition continues to face challenges due to acquired drug resistance mechanisms, including single-residue mutations such as G12D/R/V/W, G13D, Q61H, R68S, H95D/Q/R, and Y96C, as well as high expression levels of the mutated oncogene [32].

In light of these considerations, this study employed an in silico hybrid and hierarchical virtual screening protocol for the identification of novel VEGFR-2/K-RAS inhibitors. The use of our in-house ligand-based Biotarget Predictor Tool (BPT), operated in multitarget Mode, was fundamental to efficiently analyze an extensive database of active and previously optimized molecules. This advanced computational approach was further integrated with in-depth structure-based studies and molecular dynamics simulations, providing a clear example of how in silico modeling can synergistically complement experimental methodologies to accelerate the discovery of novel therapeutic agents [33].

## 2. Results and Discussion

**Figure 2:**
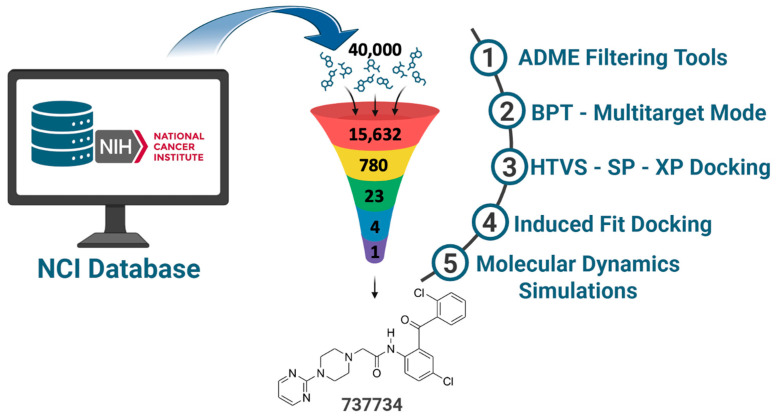
Schematic flowchart of the methodological workflow adopted in this study.

To provide a clear overview of the methodological path, we summarized the different steps of our in silico protocol in a schematic flowchart (Figure 2). The workflow began with 40,000 compounds from the NCI database, which were progressively refined by applying ADME-based filtering (QikProp and SwissADME), reducing the dataset to 15,632 drug-like molecules. These ligands were then submitted to ligand-based screening through the Biotarget Predictor Tool (BPT) in multitarget mode, which allowed us to select the top 780 candidates. Structure-based analyses were subsequently conducted through a hierarchical docking workflow, followed by Induced Fit Docking (IFD), identifying 23 dual VEGFR-2/K-RAS hits. Finally, four top-ranked molecules (**623292**, **625894**, **737734**, and **753203**) were advanced to molecular dynamics simulations for an in-depth stability assessment, alongside reference ligands for both targets.

### 2.1. NCI Database Cleaning Phase

Given the relevance of VEGFR-2 and K-RAS as promising targets for new cancer therapies, this study aims to identify potential dual-target inhibitors with anticancer and antiangiogenic properties. To this end, we screened the National Cancer Institute (NCI) database through ADME filtering tools. Our workflow started from a preliminary cleaning phase of the NCI database, selected due to its extensive collection of about 40,000 compounds, analyzed in in vitro antiproliferative assays by the National Cancer Institute against 60 cancer cell lines (NCI60). Initially, we submitted the compound library to the LigPrep tool from the Schrödinger Maestro Suite, at physiological pH (7.3 ≤ pH ≤ 7.5), to generate all possible tautomers and stereoisomers at the lowest energy state for each ligand. Subsequently, the prepared NCI database was cleaned in two consecutive steps to select small molecules with specific parameters and drug-likeness criteria.

The first step is based on the QikProp tool, which predicts the ADME (Absorption, Distribution, Metabolism, and Excretion) properties of drug candidates based on their full 3D molecular structure. QikProp enables the obtainment of a wide variety of pharmaceutically relevant parameters, including octanol/water and water/gas partition coefficients, aqueous solubility, brain/blood partition coefficient, overall Central Nervous System (CNS) activity, Caco-2 and MDCK cell permeabilities, and log Khsa for human serum albumin binding. In our approach, we focused on two specific properties: the “Rule of Five”, indicating the number of violations of the “Lipinski rule”, and #stars, a value that includes all QikProp parameters into a single index, representing the number of properties or descriptor values that are excluded from the 95% range of similar values for known drugs. A higher number of descriptors results in a higher “#stars” value, suggesting that a molecule is less drug-like than one with a lower “#stars” value. Intending to differentiate drug-like small molecules, we selected only candidate drugs with “Rule of Five” and “#star” values equal to 0, reducing the NCI database to 18,510 compounds.

To further gain insight into the drug-like nature of these compounds, we decided to delve into the analysis using the SwissADME website. This web tool allowed us to evaluate physicochemical descriptors and predict ADME parameters, pharmacokinetic properties, and the medicinal chemistry friendliness of our screened small molecules. Specifically, a set of parameters was predicted, such as the number of heavy atoms, H-bond acceptors, H-bond donors, rotatable bonds, Rule of Five, Blood–Brain Barrier (BBB) permeability, metabolic reactions, and Human Oral Absorption, which are also shared with QikProp. Additionally, our compounds were investigated concerning several criteria, including Ghose, Veber, Egan, Muegge, lead-likeness violations, bioavailability score, and PAINS alerts. Through this analysis, we chose to retain only molecules with a PAINS alert score of 0. By applying this strategy, it is possible to screen drugs already in the development phase, filtering out those with unfavorable ADME properties in clinical trials, thus optimizing the time and resources used.

In brief, to ensure selection of drug-like small molecules, compounds were filtered based on the Lipinski Rule of Five and the #stars index provided by QikProp, retaining only molecules with no violations (threshold = 0). Further filtering using SwissADME included PAINS alerts (threshold = 0) and filters as Ghose (160 ≤ MW ≤ 480; −0.4 ≤ WLOGP ≤ 5.6; 40 ≤ MR ≤ 130; 20 ≤ atoms ≤ 70), Veber (bonds ≤ 10; TPSA ≤ 140), Egan (WLOGP ≤ 5.88; TPSA ≤ 131.6), and Muegge (200 ≤ MW ≤ 600; −2 ≤ XLOGP ≤ 5; TPSA ≤ 150; Num. rings ≤ 7; Num. carbon > 4; Num. heteroatoms > 1; Num. rotatable bonds ≤ 15; H-bond acc. ≤ 10; H-bond don. ≤ 5), were applied. These thresholds were chosen based on established literature to maximize the likelihood of oral bioavailability and clinical success.

Finally, we removed the duplicates, obtaining a refined database of 15,632 drug-like small molecules, and subsequently analyzed them using the proposed in silico protocol.

### 2.2. Ligand-Based Studies

To predict the binding affinity of small molecules toward the selected biological targets (VEGFR-2 and K-RAS), in the first phase of our workflow, we employed the Biotarget Predictor Tool (BPT), a well-established, in-house-developed ligand-based protocol available on the DRUDIT platform. Identifying small molecules with activity against multiple targets represents a promising strategy for developing highly effective pharmaceutical therapies. Therefore, the refined NCI database of 15,632 ligands was screened using BPT in multitarget mode. This approach allowed us to compute the Drudit Affinity Score (DAS), which ranges from 0 to 1, where values close to 0 indicate low binding affinity and values near 1 suggest strong interactions between compounds and targets. The multitarget score (*MT-Score*) was then derived as the product of the DAS values for both targets. This approach allowed both a further reduction in the number of ligands and the specific identification of compounds with optimal activity against VEGFR-2 and K-RAS.

#### 2.2.1. Target Template Building

Using BPT required an initial phase of ligand-based template construction for the selected targets, VEGFR-2 and K-RAS. Extensive databases of known VEGFR-2 and K-RAS modulators were retrieved from BindingDB, a reliable and widely used repository of experimentally determined protein–ligand binding affinities, including K_i_, K_d_, IC_50_, and EC_50_ values, along with target information for thousands of active compounds. In this study, we applied an activity cut-off of IC_50_ < 100 nM to select the most potent inhibitors, followed by a rigorous data curation process to remove duplicate entries. This threshold ensures that only compounds with high experimental affinity for VEGFR-2 or K-RAS are included in the template construction, providing reliable molecular descriptors for the ligand-based screening and increasing the likelihood of identifying strong dual-target inhibitors. The resulting sets of active small molecules were then subjected to molecular descriptor calculation using our in-house software MOLDESTO 1.0 (Molecular Descriptor Tools), which generates over 1000 molecular descriptors (1D, 2D, and 3D) for each input structure. This process yielded a “Compounds vs. Molecular Descriptors” matrix for each dataset, which was subsequently transformed into two sequences of descriptor pairs (mean and standard deviation) to construct the molecular descriptor-based templates. These templates were incorporated into the DRUDIT 1.0 platform, enabling the evaluation of ligand affinity against them.

#### 2.2.2. Biotarget Predictor Tool—Multitarget Mode

Once templates for both targets, VEGFR-2 and K-RAS, were integrated, our ligand-based protocol included uploading the cleaned NCI database on the DRUDIT tool and investigating the affinity of small molecules contained in the dataset through BPT in multitarget mode. The affinity of every ligand toward selected biological targets is represented by the DAS, computed using the default parameter as reported [34]. DAS values for NCI compounds against both VEGFR-2 and K-RAS are available in Supplementary Material, Table S1. Given that the main aim of the study was the identification of multitarget VEGFR-2/K-RAS inhibitors, the multitarget mode was employed to calculate the *MT-Score*, defined by Equation (1):

(1) $$MT-Score={DAS}_{VEGFR-2}\times {DAS}_{K-RAS}$$

where *DAS_VEGFR-_*_2_ and *DAS_K-RAS_* indicate the DAS for each target molecular descriptor-based template, respectively. The *MT-Score* facilitated the selection of structures with optimal activity against both targets: the higher the two DAS, the higher the *MT-Score*, and consequently, the greater the likelihood of a small molecule inhibiting both targets. Compounds were ranked based on this parameter, and the top 5% (approximately 780 small molecules) were selected for further in silico investigation through structure-based studies. This threshold was chosen to balance computational efficiency with the need to retain a sufficient number of promising compounds for subsequent structure-based screening. *MT-Scores* for NCI compounds are available in Supplementary Material, Table S2.

### 2.3. Structure-Based Studies: Molecular Docking Analysis

The second phase of our protocol involved the Molecular Docking Studies, which, combined with the results obtained by the ligand-based screening, enhanced the probability of identifying small molecules capable of effectively inserting into the binding site of one or more structures of protein targets. To further filter the selected compounds in the previous phase, a two-step docking virtual screening workflow was employed. The first step utilized a protocol available in the Maestro suite, consisting of three sequential subphases of semi-flexible docking with increasing levels of accuracy, including High-throughput Virtual Screening (HTVS), Standard Precision docking (SP), and Extra Precision (XP) docking. The second step implemented Induced Fit Docking (IFD), applied to the top-ranked compounds emerging from XP docking, to assess their ability to fit into the target binding sites with even greater reliability and precision.

**Figure 3:**
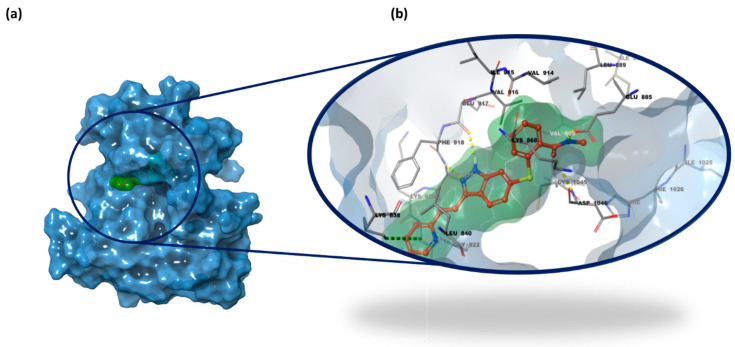
(**a**) 3D X-ray structure of VEGFR-2 HYD-I region (PDB code 4AG8 [38]); (**b**) focus on the binding pocket bound with its co-crystalized ligand **axitinib** and the main residues highlighted (PDB code 4AG8 [38]). Protein carbon atoms are shown in grey, ligand carbon atoms in red, oxygen atoms in red, nitrogen atoms in blue, and sulfur atoms in yellow.

VEGFR-2 functions as a dimer following the binding of its endogenous ligands. Its structure comprises an extracellular region with seven immunoglobulin (Ig)-like domains (I–VII), among which the second and third domains participate in VEGF ligand binding. This is followed by a transmembrane region and an intracellular portion, which includes a juxtamembrane domain and a split tyrosine kinase domain, interrupted by a 70-amino acid kinase insert and a C-terminal tail. Upon dimerization, the tyrosine kinase domain undergoes autophosphorylation. Another key event following dimerization is the exposure of the ATP-binding site, which serves as the primary target for most VEGFR inhibitors. The binding site presents a bilobed architecture, consisting of a large N-lobe and a smaller C-lobe connected by a flexible linker, which together define a front cleft and a back cleft, with the former serving as the ATP-binding site. Within this structural arrangement, the catalytic cavity of VEGFR-2 is organized into three distinct regions: the hinge region, the DFG motif, and the hydrophobic back pocket, further subdivided into HYD-I and HYD-II. The HYD-I region is enclosed by Leu840, Phe^918^, and Gly^922^, while HYD-II is defined by Leu^889^, Ile^892^, Val^898^, and Ile^1044^ amino acid residues. Its high flexibility allows the active site to adopt two distinct conformations, termed “DFG-in” (active) and “DFG-out” (inactive). Based on their binding mode to these conformations, tyrosine kinase inhibitors (TKIs) are categorized into three types (Type I, Type II, and Type III). Focusing on the first two types, Type I inhibitors bind to the “DFG-in” form by forming hydrogen bonds with key residues Cys^919^ and Glu^917^ within the hinge and HYD-I regions. Type II inhibitors, in contrast, target the “DFG-out” conformation, interacting specifically with Cys^919^ in the HYD-I region, Glu^885^ in the DFG motif region, and Asp^1046^ in the HYD-II region. Additionally, other essential residues involved in hydrogen bonding include Ile^1025^, His^1026^, and Lys^868^ [13,35,36,37]. Figure 3 illustrates the 3D binding site of VEGFR-2 in complex with compound **axitinib** (PDB code 4AG8 [38]).

**Figure 4:**
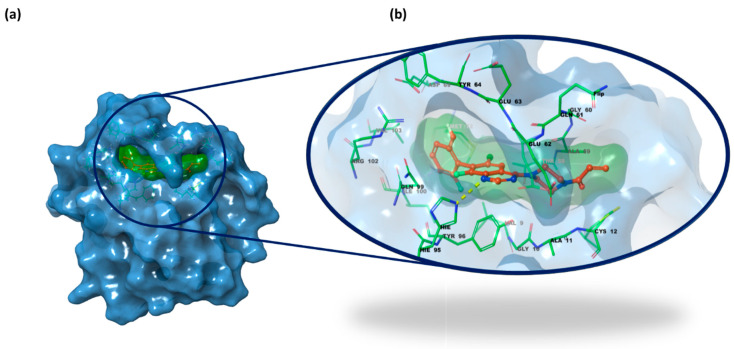
(**a**) X-ray structure of K-RAS switch II region of G domain (PDB code 5V9U [42]); (**b**) focus on the switch II pocket bound with its co-crystalized ligand ***ARS-1620*** and the main residues highlighted (PDB code 5V9U [42]). Protein carbon atoms are shown in grey, ligand carbon atoms in green, oxygen atoms in red, nitrogen atoms in blue, and sulfur atoms in yellow.

On the other hand, the structure of K-RAS consists of six β-strands and five α-helices, which together form two main domains: the catalytic G domain (GTP binding domain) and the hypervariable region (HVR). The G domain is further divided into three key regions: switch I, switch II, and the P-loop, which interact with guanine nucleotides to regulate signaling through ligand binding. The HVR contains the CAAX motif, which plays a crucial role in anchoring K-RAS to the membrane. The current findings allowed for a more precise characterization of the protein’s binding sites beyond the nucleotide-binding domain, identifying multiple pockets in proximity to the switch I and switch II regions. Specifically, these include the switch II pocket, the switch I/II pocket, and the A59 site. Among these binding cavities, the primary focus of our investigation on the K-RAS^G12C^ mutant was the switch II pocket, which is defined by the amino acid residues Glu^63^, Asp^69^, Met^72^, and Tyr^96^. The primary amino acid residues involved in K-RAS inhibitor binding include Cys^12^ within the switch II region binding pocket, where small molecules can form covalent bonds. Additionally, hydrogen bonding interactions with Lys^16^, Asp^69^, and His^95^ play a stabilizing role in the K-RAS–inhibitor complex, further enhancing binding affinity [19,39,40,41]. Figure 4 depicts K-RAS G12C in a complex with compound **ARS-1620** (PDB code 5V9U [42]).

Once docking grids were centered on the VEGFR-2 and K-RAS binding sites, a virtual screening workflow, including HTVS, SP, and XP docking, was performed on a set of 780 small molecules. At each stage, the top 50% of ranked compounds were selected for the subsequent docking analysis, ultimately yielding 97 ligands, among which 23 were found to be common to both targets (results from the VSW for both VEGFR-2 and K-RAS are available in Supplementary Material, Table S3). Standard molecular docking studies typically assume a rigid receptor; however, receptors undergo conformational adjustments to accommodate ligand binding. To account for this, the Induced Fit Docking (IFD) protocol was implemented, enabling a more detailed analysis of the structural characteristics of ligand-target complexes and their conformational changes.

**Figure 5:**
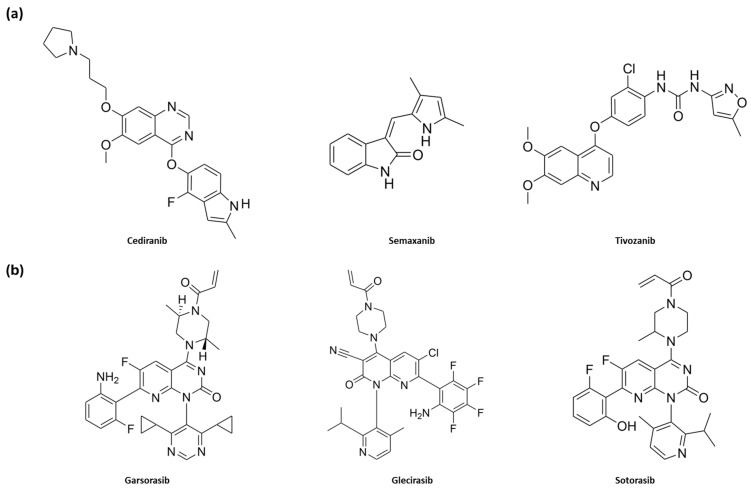
(**a**) 2D chemical structures of reference compounds **cediranib**, **semaxanib,** and **tivozanib** for VEGFR-2 (for 2D chemical structure of compound **axitinib** see Figure 1); (**b**) 2D chemical structures of reference compounds **garsorasib**, **glecirasib,** and **sotorasib** for K-RAS (for 2D chemical structure of compounds **adagrasib** and **ARS-1620** see Figure 1).

In the second step of the docking study, the IFD protocol was applied using the same X-ray structures depicted in Figure 3 and Figure 4, while the IFD scores for the 23 selected small molecules are reported in Table 1. In this analysis, we included a validation set of 9 compounds as well-known inhibitors of VEGFR-2 and K-RAS (**axitinib**, **cediranib**, **semaxanib**, and **tivozanib** for VEGFR-2; **adagrasib**, **garsorasib**, **glecirasib**, **sotorasib**, and **ARS-1620** for K-RAS), illustrated in Figure 5.

**Figure 6:**
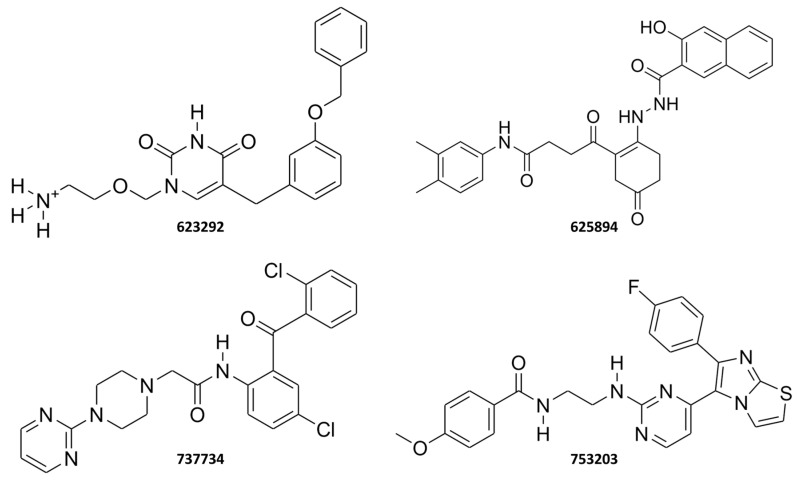
Two-dimensional chemical structures of compounds **623292**, **625894**, **737734**, and **753203**.

**Figure 7:**
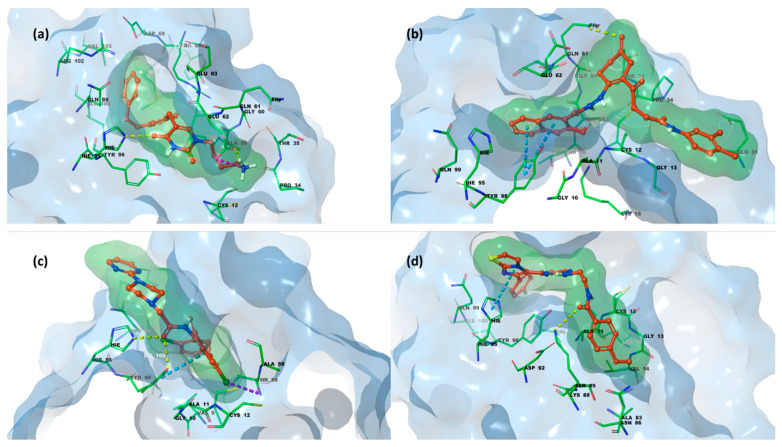
(**a**) 3D x-ray structure of K-RAS switch II domain in complex with compound **623292**; (**b**) 3D structure of K-RAS in complex with compound **625894**; (**c**) 3D structure of K-RAS in complex with compound **737734**; (**d**) 3D structure of K-RAS in complex with compound **753203**. Protein carbon atoms are shown in green, ligand carbon atoms in red, oxygen atoms in red, nitrogen atoms in blue, and sulfur atoms in yellow.

**Figure 8:**
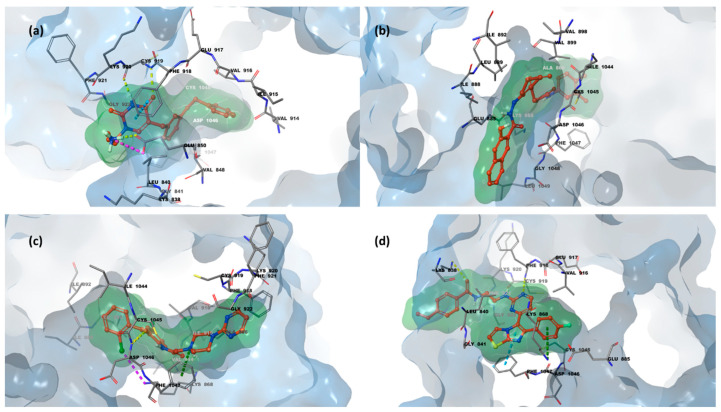
(**a**) 3D x-ray structure of VEGFR-2 binding pocket in complex with compound **623292**; (**b**) 3D structure of VEGFR-2 in complex with compound **625894**; (**c**) 3D structure of VEGFR-2 in complex with compound **737734**; (**d**) 3D structure of VEGFR-2 in complex with compound **753203**. Protein carbon atoms are shown in grey, ligand carbon atoms in red, oxygen atoms in red, nitrogen atoms in blue, and sulfur atoms in yellow.

The results of the IFD simulations revealed that several compounds exhibited strong interaction capabilities with both biological targets, achieving IFD scores that were either higher or comparable to those of the reference ligands. Notably, compounds **753203**, **737734**, **625894**, and **623292**, listed among the 23 molecules reported in Table 1, displayed the best IFD scores for both proteins. Respectively, for K-RAS and VEGFR-2, the following IFD scores were obtained: **753203** (−464.212 and −669.154), **737734** (−462.348 and −668.884), **625894** (−462.446 and −663.527), and **623292** (−462.176 and −664.999). Furthermore, in the case of K-RAS, all four compounds demonstrated superior scores compared to the well-known inhibitors **garsorasib**, **glecirasib**, and **sotorasib**. Similarly, for VEGFR-2, **753203** and **737734** ligands outperformed the co-crystallized ligand **axitinib**, as well as the reference inhibitors **cediranib** and **tivozanib** included in the validation set, with a significant margin achieved over **semaxanib** even for **625894** and **623292**. The 2D structures of these four promising small molecules are depicted in Figure 6, while Figure 7 and Figure 8 illustrate the 3D complexes formed by compounds **623292**, **625894**, **737734**, and **753203** within the binding sites of K-RAS and VEGFR-2, respectively.

In Figure 7, the crucial role of His^95^, previously discussed, is highlighted in mediating the interaction between compounds **623292**, **625894**, **737734**, and **753203** and the switch II domain, which constitutes the allosteric binding site of K-RAS. Specifically, for compounds **623292** and **737734**, His^95^ is involved in the formation of a hydrogen bond; in the case of compound **753203**, it participates in a π–π stacking interaction with the imidazole ring of the candidate small molecule.

In Figure 8, the key role of several previously mentioned amino acid residues is evident in mediating the interactions between compounds **623292**, **625894**, **737734**, and **753203** and the HYD-I and HYD-II regions, which constitute the binding sites of VEGFR-2. For compounds **623292** and **753203**, the residue Cys^919^ is involved in the formation of an H-bond, while for compound **737734**, Asp^1046^ is the residue responsible for establishing an H-bond.

Therefore, based on the IFD scores achieved by compounds **623292**, **625894**, **737734**, and **753203**, additional investigations were carried out through Molecular Dynamics Simulations to gain deeper insights into the interactions of these four ligands when complexed with K-RAS and VEGFR-2.

### 2.4. Molecular Dynamics Simulations

Molecular Dynamics Simulations were performed for compounds **623292**, **625894**, **737734**, and **753203**, as well as for their respective reference drugs previously mentioned, in complex with K-RAS and VEGFR-2, to reproduce the dynamic behavior of the model systems by generating the trajectories of the individual atoms and molecules during the simulation time.

In the initial phase, all selected compounds were subjected to 50 ns simulations, providing a consistent temporal window to extract relevant and meaningful structural and energetic data. The Root Mean Square Deviation (RMSD) was computed for both ligands and proteins throughout the trajectory of simulations lasting 50 ns for each ligand-protein complex. The analysis aimed to evaluate the stability and convergence of the simulations by measuring the average change in displacement of the backbone from a reference frame at t = 0. RMSD provides a measure of the structural deviation across all frames when compared to the reference structure at the beginning of the simulation. A stabilized RMSD value that only fluctuates around a mean value confirms the equilibration of the system and the convergence of the simulation. Fluctuations in the order of 1 to 3 Å are expected for small globular proteins, while larger fluctuations may indicate conformational changes.

**Figure 9:**
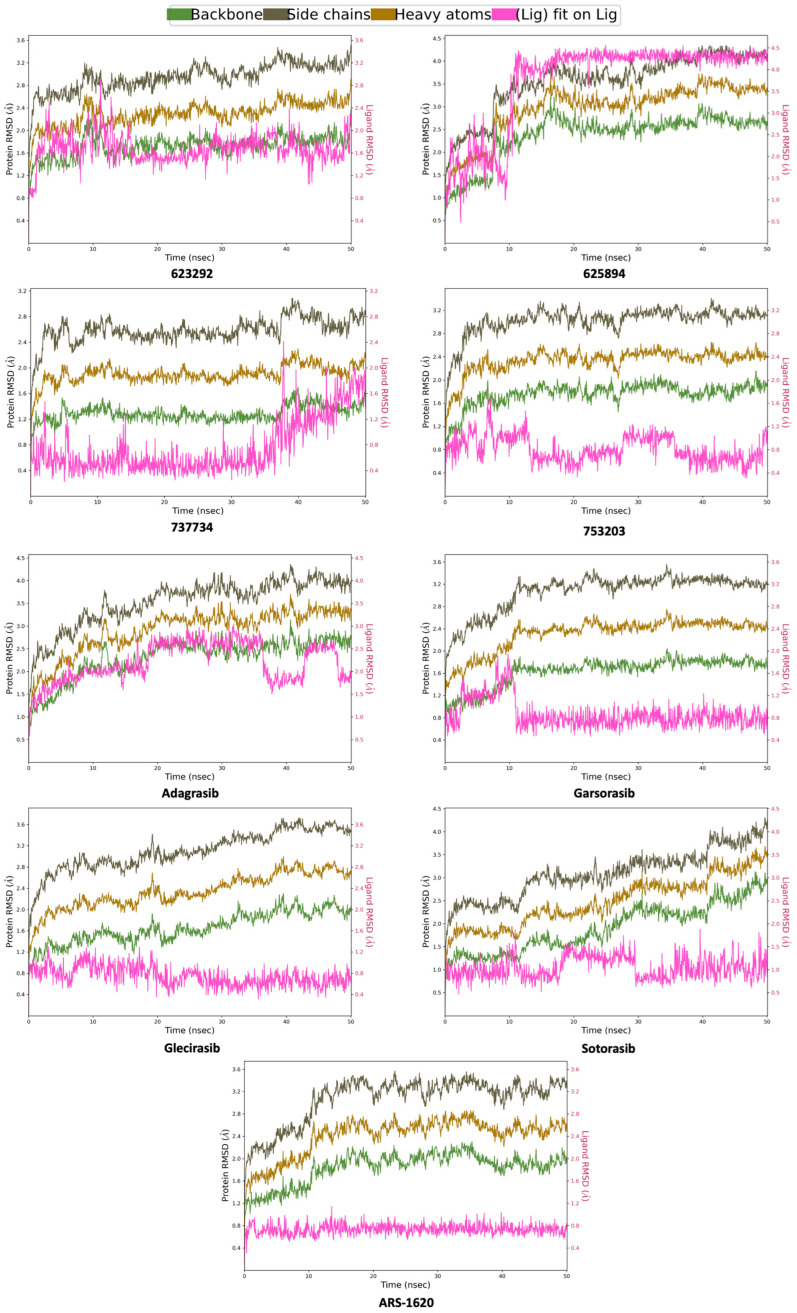
Computed RMSD for K-RAS protein backbone, side chains, heavy atoms, and ligand aligned to the ligand itself for compounds **623292**, **625894**, **737734**, and **753203**, and the reference ligands (**adagrasib**, **garsorasib**, **glecirasib,** and **sotorasib**) and ligand co-crystalized **ARS-1620**, during the simulation trajectory of 50 ns.

According to the RMSD analysis for K-RAS protein, depicted in Figure 9, almost all compounds demonstrate a comparable RMSD to the reference ligands (**adagrasib**, **garsorasib**, **glecirasib**, **sotorasib**, and **ARS-1620**). In the conducted simulations, both the protein and the ligands achieved satisfactory equilibration, with no evidence of significant conformational rearrangements. As expected, the protein side chains exhibited higher RMSD values due to their greater flexibility relative to the more rigid protein backbone. The ligand RMSD, calculated by aligning each frame to the initial ligand conformation, reflects internal fluctuations within the ligand structure. In this context, no substantial conformational alterations were observed.

**Figure 10:**
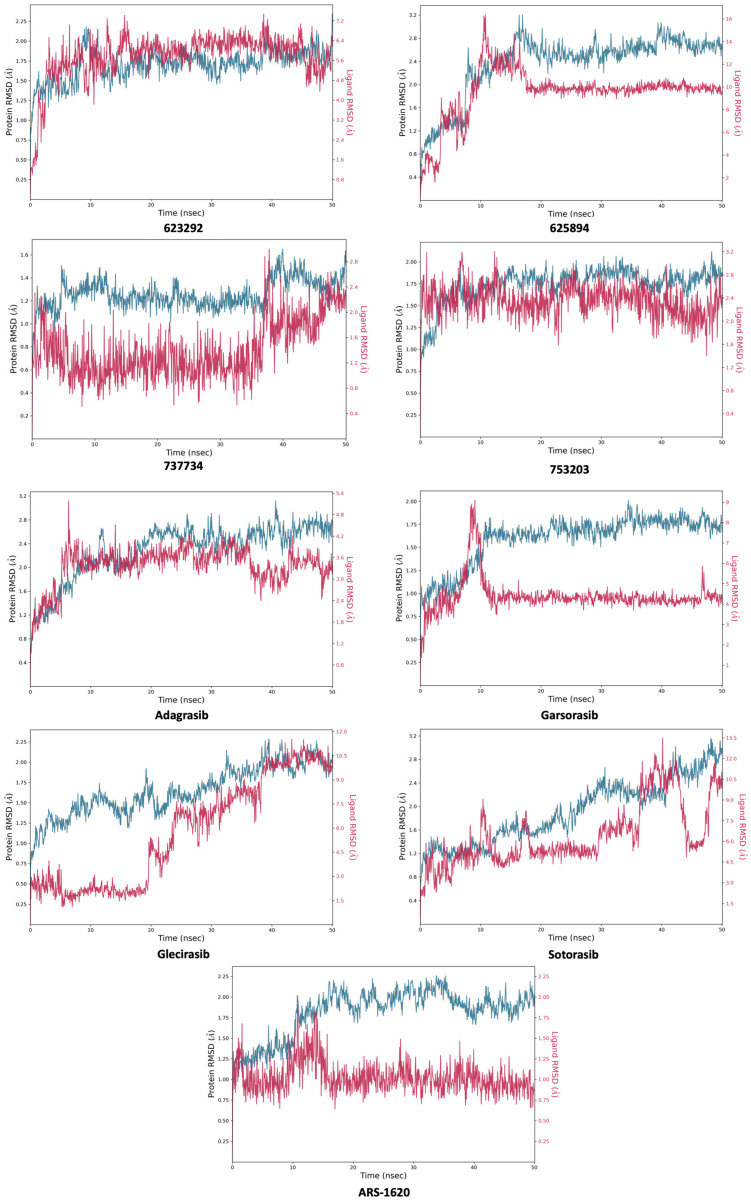
Calculated RMSD for K-RAS protein C-alphas and ligand aligned to the protein for compounds **623292**, **625894**, **737734**, and **753203**, and the reference ligands (**adagrasib**, **garsorasib**, **glecirasib**, **sotorasib,** and **ARS-1620**), during the simulation trajectory of 50 ns.

Figure 10 displays the ligand RMSD values aligned to the protein, which indicate the positional fluctuations of the ligand with respect to the protein throughout the simulation. Notably, only compounds **737734** and **753203** exhibited favorable outcomes, displaying superior stability not only when compared to the other two candidate molecules, **623923** and **625894**, which reached RMSD peaks of 7.2 and 16 Å, respectively, but also relative to the four reference inhibitors (**adagrasib**, **garsorasib**, **glecirasib**, and **sotorasib**); in particular, **garsorasib**, **glecirasib**, and **sotorasib** recorded maximum RMSD values of 9, 12, and 13.5 Å, respectively, further confirming the greater stability of the complexes formed by **737734** and **753203** with K-RAS.

**Figure 11:**
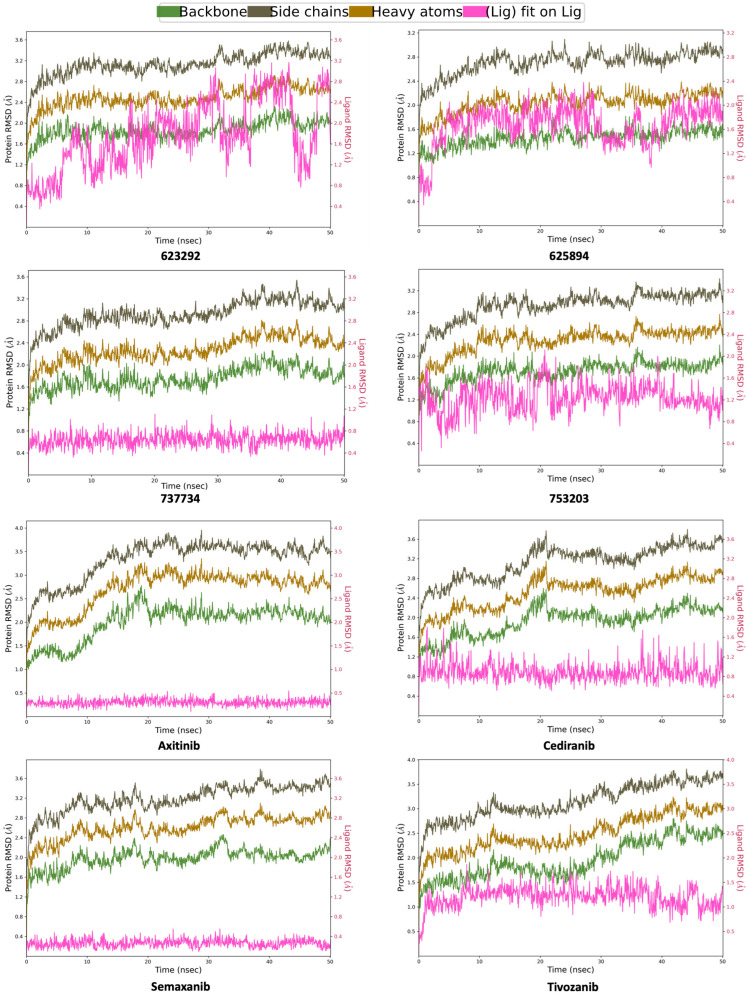
Computed RMSD for VEGFR-2 protein backbone, side chains, heavy atoms, and ligand aligned to the ligand itself for compounds **623292**, **625894**, **737734**, and **753203**, and the reference ligands (**axitinib**, **cediranib**, **semaxanib**, and **tivozanib**), during the simulation trajectory of 50 ns.

Subsequently, RMSD analysis was also performed for the same candidate compounds in complex with VEGFR-2, alongside the respective reference ligands (**axitinib**, **cediranib**, **semaxanib**, and **tivozanib**), maintaining the initial simulation trajectory of 50 ns for all systems. From the analysis of the graphs shown in Figure 11, it is evident that both the protein and the ligands reached satisfactory equilibration, without showing any relevant conformational rearrangements. The protein RMSD remained within a range of 1 to 3.5 Å, while all compounds exhibited ligand RMSD values aligned to the ligand itself within acceptable limits, indicating the absence of significant conformational changes.

**Figure 12:**
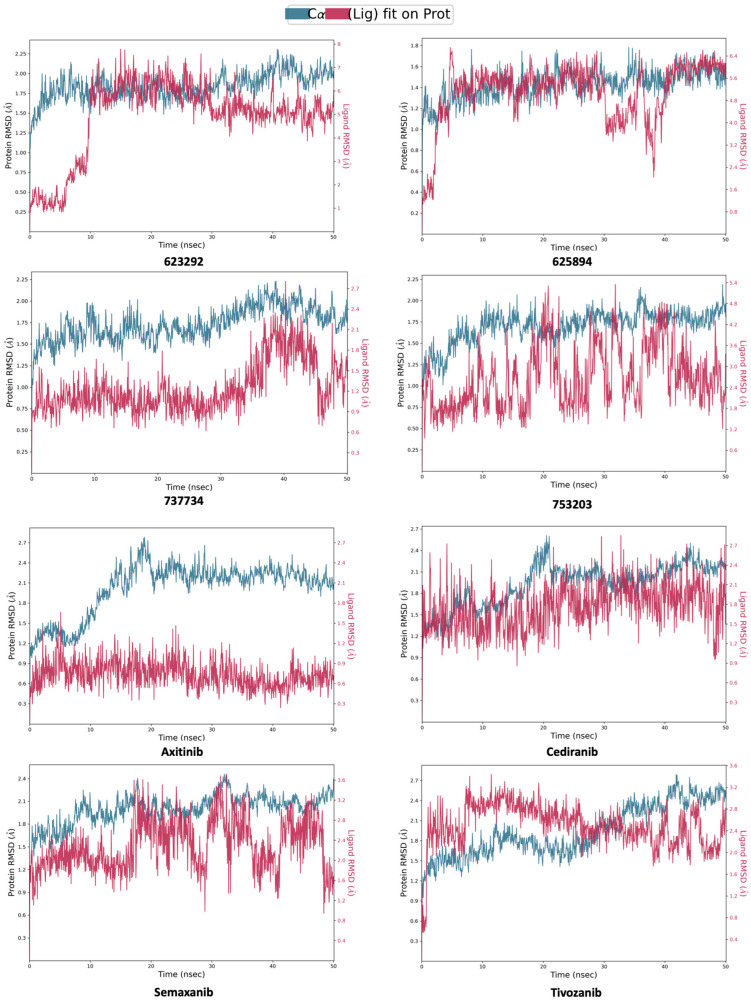
Calculated RMSD for VEGFR-2 protein C-alphas and ligand aligned to the protein for compounds **623292**, **625894**, **737734**, and **753203**, and the reference ligands (**axitinib**, **cediranib**, **semaxanib**, and **tivozanib**), during the simulation trajectory of 50 ns.

As shown in Figure 12, the ligand RMSD aligned to the protein revealed unsatisfactory results for compounds **623292** and **625894**, with peak deviations of 8.0 Å and 6.4 Å, respectively. On the other hand, the analysis confirmed that compounds **737734** and **753203** maintained acceptable RMSD values, indicating a good level of convergence between the ligand and the protein. Specifically, compound **737734** exhibited particularly stable behavior, reflecting the optimal stability of the complex formed with VEGFR-2.

**Figure 13:**
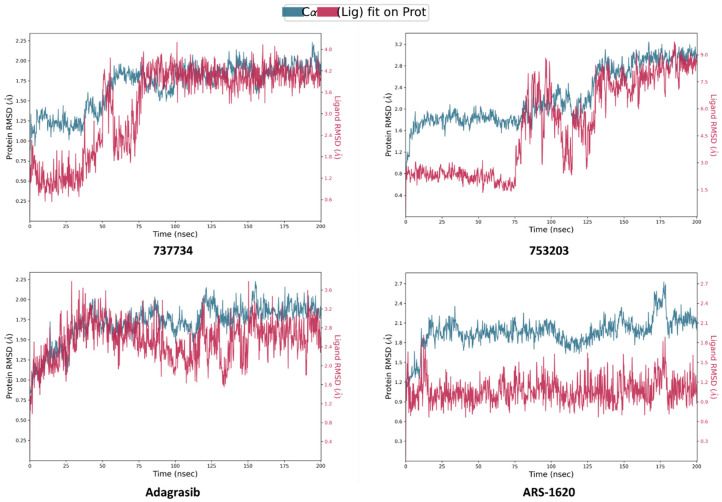
Calculated RMSD for K-RAS protein C-alphas and ligand aligned to the protein for compounds **737734** and **753203**, and the reference ligands (**adagrasib** and **ARS-1620**), during the simulation trajectory of 200 ns.

**Figure 14:**
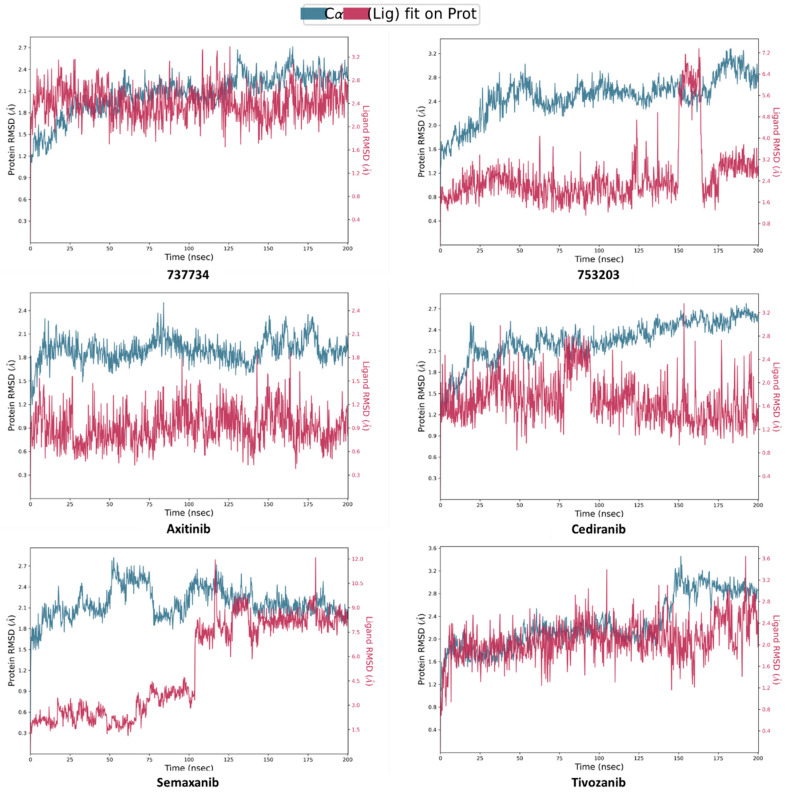
Calculated RMSD for VEGFR-2 protein C-alphas and ligand aligned to the protein for compounds **737734** and **753203**, and the reference ligands (**axitinib**, **cediranib**, **semaxanib**, and **tivozanib**), during the simulation trajectory of 200 ns.

In light of these findings, we deemed it appropriate to conduct a further investigation by extending the RMSD analysis over a longer simulation timescale, to confirm the stability of the interactions and detect any structural variations not evident within the initial 50 ns. This extended analysis provided the opportunity to observe the long-term dynamic behavior of the selected compounds from previous simulations, especially for **737734** and **753203**, which have achieved good results associated with both proteins. Simultaneously, reference ligands exhibiting less favorable RMSD profiles—particularly in the case of K-RAS, such as **garsorasib**, **glecirasib**, and **sotorasib**—were excluded from further consideration. Figure 13 and Figure 14 illustrate the RMSD profiles for compounds **737734** and **753203**, both complexed with K-RAS and VEGFR-2, respectively, over a 200 ns simulation period.

In particular, the graphs shown in Figure 13 illustrate that, over a prolonged simulation timescale, compound **753203** exhibits an RMSD profile reaching peaks of 9.0 Å, which exceeds the RMSD observed for the protein itself, indicating that the stability previously observed in complex with K-RAS during the 50 ns simulation is not maintained at 200 ns; conversely, compound **737734** displays an RMSD value comparable to those of the reference ligands, supporting the sustained stability of its interaction with the target.

**Figure 15:**
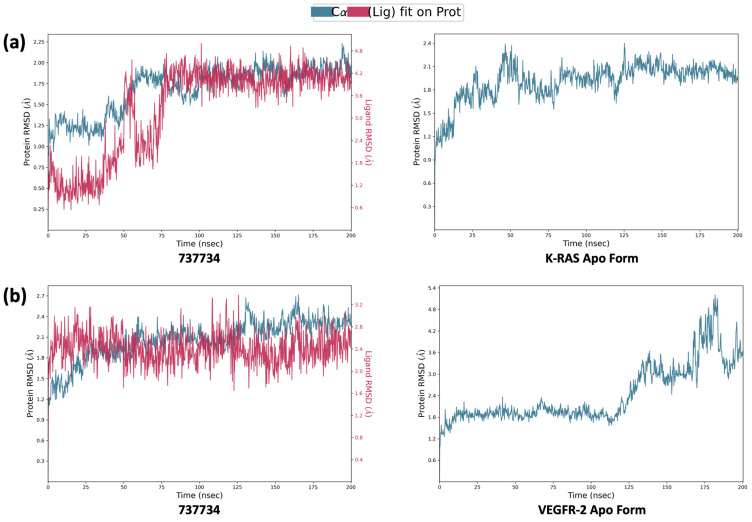
(**a**) Calculated RMSD for K-RAS protein C-alphas and ligand aligned to the protein for compound **737734**, and respective RMSD for K-RAS protein C-alphas as apo form, during the simulation trajectory of 200 ns; (**b**) calculated RMSD for VEGFR-2 protein C-alphas and ligand aligned to the protein for compound **737734**, and respective RMSD for VEGFR-2 protein alphas as apo form, during the simulation trajectory of 200 ns.

The graphs in Figure 14 confirm that compound **737734** exhibits greater stability not only in comparison with compound **753203** but also with the well-known drug ***semaxanib***, which reaches RMSD peaks of 7.2 and 12 Å, respectively. These findings suggest that the selected compound **737734** maintains a robust RMSD profile at both protein binding sites, indicating its ability to establish strong and stable interactions with K-RAS and VEGFR-2, thereby reinforcing its potential as a dual inhibitor of both molecular targets. Additionally, a further molecular dynamics simulation was performed over a 200 ns timescale to compare the RMSD profile of the selected compound **737734** with that of the apo form of each protein, defined as the protein in the absence of a bound ligand at its binding site; the results of this analysis, illustrated in Figure 15, show that, when comparing the protein RMSD of the complexes formed between **737734** and both biological targets, K-RAS and VEGFR-2, with the RMSD profiles of the respective apo forms, the complexes appear more stable. Specifically, for K-RAS, the RMSD of the complex is 2.25 Å compared to 2.4 Å for the apo form, while for VEGFR-2, the difference is more pronounced, with an RMSD of 2.7 Å for the complex versus 5.4 Å for the unbound protein.

Subsequently, to extract additional meaningful molecular features, a comprehensive analysis of various structural parameters was conducted for each previously analyzed complex over a 200 ns simulation. This included the calculation of the Ligand Root Mean Square Fluctuation (L-RMSF) and Protein Ligand contacts. Radius of Gyration (rGyr), which assesses the “extendedness” of the ligand and correlates with its principal moment of inertia, Intramolecular Hydrogen Bonds (intraHB), Molecular Surface Area (MolSA), Solvent Accessible Surface Area (SASA), and Polar Surface Area (PSA), were also analyzed and provided in Supplementary Material, Figures S1–S5.

**Figure 16:**
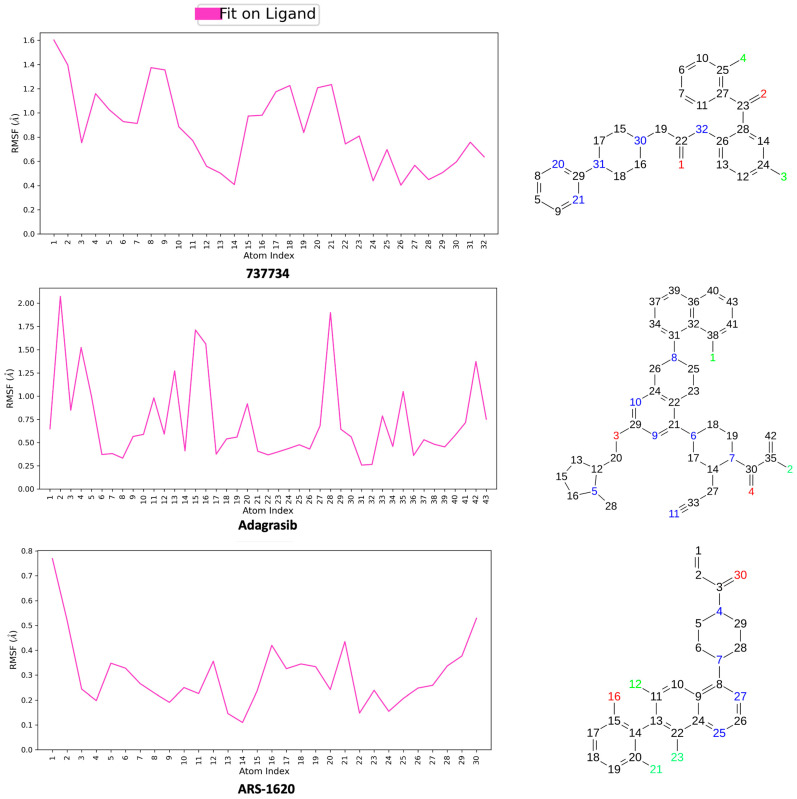
Calculated L-RMSF for compounds **737734**, **adagrasib**, and **ARS-1620** when in complex with K-RAS, during the simulation trajectory of 200 ns alongside 2D structures of related ligands.

**Figure 17:**
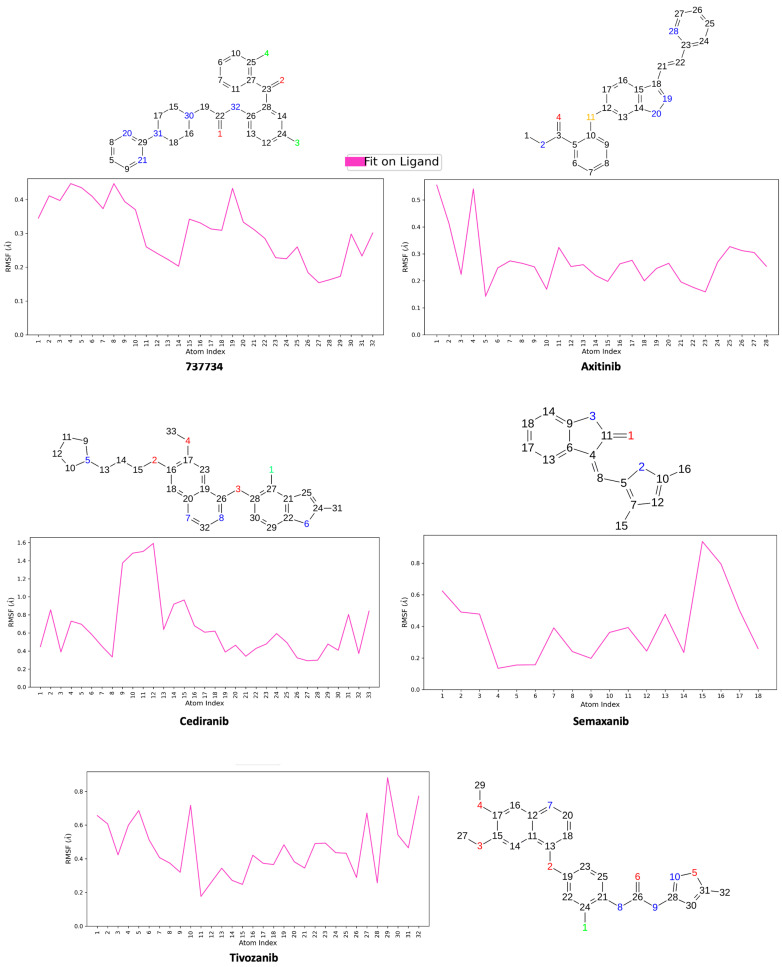
Calculated L-RMSF for compounds **737734**, **axitinib**, **cediranib**, **semaxanib**, and **tivozanib** when in complex with VEGFR-2, during the simulation trajectory of 200 ns alongside 2D structures of related ligands.

The Ligand Root Mean Square Fluctuation (L-RMSF) represents a key parameter for evaluating the atomic mobility of the ligand, enabling the identification of regions with significant positional deviations over time. In our simulations, L-RMSF analysis was performed for compound **737734** within each complex formed with K-RAS and VEGFR-2, using as reference compounds **adagrasib** and **ARS-1620** for K-RAS, and **axitinib**, **cediranib**, **semaxanib**, and **tivozanib** for VEGFR-2, respectively. The reference time (t_ref_) was defined as the first frame of the simulation, establishing it as the temporal zero point. The L-RMSF values, shown in Figure 16 and Figure 17, provide valuable information on the local flexibility of individual atoms. The ligand was aligned to its reference frame, and the RMSF was calculated for heavy atoms only. A 2D representation of each molecule is displayed above the corresponding plot to indicate atom number, name, and type. As shown in Figure 16, the atoms exhibiting the highest RMSF values in compound **737734** are the oxygen atoms, while all remaining atoms display relatively low fluctuations, with RMSF values ranging from 0 to 1.4 Å.

In Figure 17, concerning the VEGFR-2 complex, the atoms showing higher RMSF values in compound **737734** are the chlorine atoms; additionally, the external portion of the pyrimidine ring, as well as the C-alpha atom labeled as 19, displays a certain degree of flexibility. All remaining atoms show low RMSF values, ranging from 0 to 0.4 Å. These results demonstrate that the candidate compound **737734** exhibits slightly greater stability in complex with VEGFR-2 compared to K-RAS.

### 2.5. Integrated Analysis of Amino Acid Interactions Between Compound **737734** and Protein Binding Sites

Based on the findings from molecular dynamics simulations, we conducted a detailed analysis of the key interactions between the best docked pose of compound **737734** and the amino acid residues within each protein binding site (Table 2). This analysis aims to compare the protein–ligand interactions observed in IFD studies, which provide a detailed snapshot of the static interactions in the optimal binding configuration, with those captured during molecular dynamics simulations, which provide insights into the stability and dynamic behavior of these interactions over time. This integrated approach allows for a comprehensive understanding of ligand-binding interactions.

The derivative **737734** exhibited a comparable number of interactions with the co-crystalized ligands **ARS-1620** and **axitinib**, within a 4 Å proximity.

Within the K-RAS binding pocket, compound **737734** established multiple interactions with key residues of the active site.

As previously described, the switch II pocket plays a crucial role in ligand binding and comprises several essential amino acid residues, including Cys^12^, Lys^16^, Glu^63^, Asp^69^, Met^72^, His^95^, and Tyr^96^. Among these, His^95^ formed a hydrogen bond between the hydrogen of the imidazole ring in its side chain and the oxygen of the carbonyl group in compound **737734** (N-H---O). Tyr^96^ contributed an additional hydrogen bond through the hydroxyl group of its phenol ring to the same carbonyl oxygen (O-H---O), and simultaneously engaged in a π–π stacking interaction. Furthermore, a halogen bond was observed between the chlorine atom and the carbonyl oxygen of Thr^58^. Notably, two water-mediated bridges were also identified: the first involving Glu^63^ and the carbonyl oxygen, and the second connecting Arg^102^ with the nitrogen atom of the pyrimidine ring of the compound **737734**.

**Figure 18:**
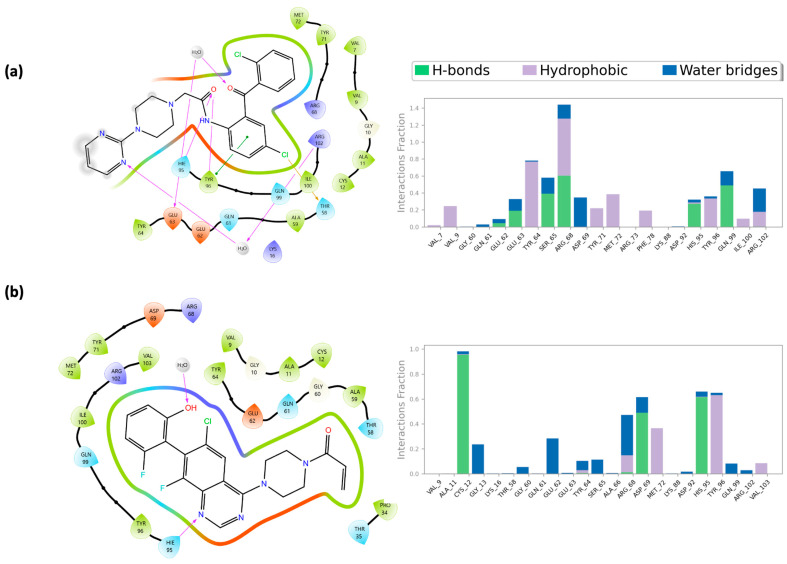
(**a**) The two-dimensional structure of the detailed interactions between the atoms of ligand **737734** and the protein residues of K-RAS and protein–ligand interactions examination across the simulation time for **737734**/K-RAS complex; (**b**) two-dimensional structure of the detailed interactions between the atoms of co-crystalized ligand **ARS-1620** and the protein residues of K-RAS and protein–ligand interactions examination across the simulation time for **ARS-1620**/K-RAS complex. On the left side, hydrogen bonds are labeled in purple, while π–π stacking in green.

A comparison of these findings, also represented in Figure 18 through 2D interaction maps, with the dynamic protein–ligand interactions derived from molecular dynamics simulations, likewise illustrated in Figure 18, highlights the interaction fraction of protein residues with the ligand, indicating how consistently specific interactions are maintained within the receptor-ligand complexes throughout the simulation time. Specifically, Figure 18a,b reports the protein–ligand contact profiles for compounds **737734** and ***ARS-1620*** in complexes with K-RAS. Notably, the candidate compound **737734** exhibited a comparable or even superior interaction fraction with key residues such as Tyr^64^, Ser^65^, Arg^68,^ and Gln^99^ during the entire trajectory, whereas the reference ligand ***ARS-1620*** maintained lower interaction fractions with residues Cys^12^, Asp^69^, His^95^, and Tyr^96^.

For VEGFR-2 as well, compound **737734** displayed the key functional groups necessary to accommodate the catalytic site of the protein. Specifically, compound **737734** was involved in the formation of a hydrogen bond between the amide hydrogen of the Asp^1046^ residue and the carbonyl oxygen of the ligand. Additionally, a halogen bond was observed between the chlorine atom of the ligand and the hydrogen of a positively charged quaternary nitrogen atom of Lys^868^. A π-cation interaction was also detected between the aromatic ring of Phe^1047^ and the same positively charged nitrogen. Finally, a water bridge interaction was established between Leu^840^ and the nitrogen atom of the pyrimidine ring of compound **737734**.

**Figure 19:**
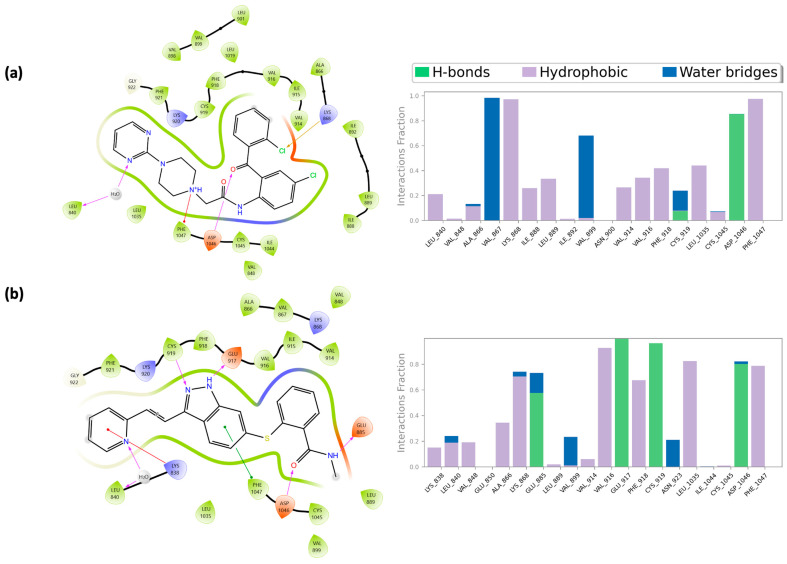
(**a**) The two-dimensional structure of the detailed interactions between the atoms of ligand **737734** and the protein residues of VEGFR-2 and protein–ligand interactions examination across the simulation time for **737734**/VEGFR-2 complex; (**b**) two-dimensional structure of the detailed interactions between the atoms of co-crystalized ligand **axitinib** and the protein residues of VEGFR-2 and protein–ligand interactions examination across the simulation time for **axitinib**/VEGFR-2 complex. On the left side, hydrogen bonds are labeled in purple, while π–π stacking in green.

Also in this case, Figure 19 compares the 2D interaction maps with the dynamic protein–ligand interactions derived from molecular dynamics simulations. Specifically, Figure 19 illustrates the protein–ligand contact profiles for compounds **737734** and **axitinib** in complex with VEGFR-2. Notably, the candidate compound **737734** demonstrated comparable, or in some cases superior, interaction fractions with key residues such as Val^867^, Ly^s868^, Val^899^, Asp^1046^, and Phe^1047^ throughout the entire simulation trajectory, whereas the reference ligand **axitinib** maintained consistently lower interaction fractions with the same residues.

The Molecular Mechanics with Generalized Born and Surface Area (MM-GBSA) method was applied to calculate the ligand binding energies of compound **737734** and the reference ligands **ARS-1620** and **axitinib** for their respective biological targets, K-RAS and VEGFR-2. This method employs an implicit solvent model to account for the desolvation effects of both ligand and receptor. For the calculations, the VSGB water model was used to simulate solvation. A comparison of the energy values (Table 3) between compound **737734** and the ARS-1620/K-RAS complex provides insight into the relative binding affinities, revealing that the candidate compound exhibited energy values comparable to the well-known drug. In detail, compound **737734** exhibits: Prime Coulomb Energy (−6302.17), Prime vdW Energy (−851.78), Prime Solvent Energy (−1609.49), Prime Hbond Energy (−133.84), Prime Energy (−9040.7); while ***ARS-1620*** demonstrates Prime Coulomb Energy (−6305.19), Prime vdW Energy (−847.79), Prime Solvent Energy (−1626.10), Prime Hbond Energy (−133.56), and Prime Energy (−9063.7). Similarly, Table 3 presents the comparison of energy values between compound **737734** and the ***axitinib***/VEGFR-2 complex, highlighting that the candidate compound exhibited even more favorable energy values than the reference ligand. Specifically, compound **737734** recorded: Prime Coulomb Energy (−9172.15), Prime vdW Energy (−1549.90), Prime Solvent Energy (−1824.98), Prime Hbond Energy (−142.24), and Prime Total Energy (−13,112.3); whereas ***axitinib*** displayed Prime Coulomb Energy (−9151.63), Prime vdW Energy (–1578.12), Prime Solvent Energy (−1744.62), Prime Hbond Energy (−145.96), and Prime Total Energy (−13,056.9). These results further support the consistent superior performance of compound **737734** across all the molecular simulations conducted.

## 3. Materials and Methods

### 3.1. Database Cleaning Phase

In this study, we employed two well-established computational tools, QikProp (Release 2017-1; Schrödinger LLC, New York, NY, USA), and SwissADME (https://www.swissadme.ch/, Swiss Institute of Bioinformatics, Lausanne, Switzerland; accessed on 25 March 2025), to predict the pharmacokinetic profiles of the designed compounds and to identify those lacking drug-like characteristics. QikProp [2], a core module within the Schrödinger Suite (Maestro Version 11.1.012, Release 2017-1), utilizes molecular descriptors to estimate a range of drug-like properties, including solubility, membrane permeability, and oral bioavailability. Default settings were employed for calculations, yielding insights into the compounds’ ADME (Absorption, Distribution, Metabolism, and Excretion) properties. Moreover, we utilized SwissADME [43], a web-based tool developed by the Swiss Institute of Bioinformatics, to further assess the pharmacokinetic parameters. SwissADME enables the evaluation of key physicochemical descriptors and the prediction of ADME parameters, pharmacokinetic behavior, and medicinal chemistry suitability properties. The integration of QikProp and SwissADME analyses provided a comprehensive understanding of the potential drug-like characteristics of the investigated compounds, facilitating the selection of lead candidates for subsequent experimental validation.

### 3.2. Ligand-Based Studies

DRUDIT [44] operates on four servers, each capable of concurrently handling more than ten jobs. These servers run various software modules implemented in C (version 11) and JAVA (version 8, Oracle Corporation, Austin, TX, USA) on MacOS Mojave (version 10.14; Apple Inc., Cupertino, CA, USA). Specifically, the Biotarget Predictor Tool (BPT, Palermo, Italy) was employed in a multitarget mode to screen the extensive, refined NCI database of active ligands for potential VEGFR-2 and K-RAS dual inhibitors.

The Biotarget Predictor Tool (BPT) enables the prediction of binding affinity between candidate compounds and selected biological targets. Target templates for VEGFR-2 and K-RAS were generated using datasets of well-characterized protein inhibitors with binding affinities below 100 nM, retrieved from BindingDB. Molecular docking simulations were subsequently performed at the corresponding binding sites to accurately orient the ligands within the active pockets. The templates were incorporated into DRUDIT, employing default parameters [44]. Descriptor selection and scoring were carried out according to the DRUDIT Affinity Score (DAS) algorithm, which depends on three user-defined parameters: N, Z, and G. Specifically, N defines the number of dynamically selected molecular descriptors, Z sets the maximum allowed percentage of unavailable (zero) values per descriptor, and G controls the Gaussian smoothing function used for descriptor weighting. In this study, the following parameter values were employed: N = 500, Z = 50, and G = a. Descriptors were ranked according to their statistical relevance toward each biological target, and only the top N descriptors meeting the Z threshold were retained. The final DAS value was computed as the weighted mean of the scores assigned by the Gaussian function (G) to the selected molecular descriptors, ensuring balanced representation and reproducibility of the computed affinity scores.

In the initial phase of the in silico workflow, the meticulously cleaned NCI database was uploaded to DRUDIT and subjected to the Biotarget Predictor in a multitarget mode. The output results yielded a DAS value for each structure, representing the binding affinity of compounds against the targets.

In detail, the “Multi-Target Score” was computed with Equation (1).

### 3.3. Structure-Based Studies

The preparation of ligands and proteins for in silico studies was carried out following the subsequent rigorous, detailed procedures:

#### 3.3.1. Ligand Preparation

The ligands designated for docking were prepared using the LigPrep tool (Release 2017-1; Schrödinger LLC, New York, NY, USA) within the Schrödinger Maestro Suite [45]. Each ligand underwent exhaustive tautomer and stereoisomer generation at a pH of 7.0 ± 0.4, employing default settings and the Epik ionization method [46]. Following this, the Optimized Potentials for Liquid Simulations (OPLS 2005) force field was employed to minimize the energy status of the ligands [47].

#### 3.3.2. Protein Preparation

Crystal structures of VEGFR-2 and K-RAS (PDB codes 4AG8 [38] and 5V9U [42], respectively) were retrieved from the Protein Data Bank [48,49]. Using the Protein Preparation Wizard (Release 2017-1; Schrödinger LLC, New York, NY, USA) within the Schrödinger software suite, default settings were applied to prepare these structures [50]. The protocol included the assignment of bond orders, retention of Het groups, removal of crystallographic water molecules, and adjustment of protonation states of heteroatoms using Epik, at a biologically relevant pH (7.0 ± 0.4). Hydrogen bond networks were then optimized, followed by a restrained energy minimization using the OPLS 2005 force field, with a Root Mean Square Deviation (RMSD) cut-off for atomic displacement set to 0.3 Å [47].

The OPLS force field series, particularly OPLS-2005, is specifically developed and parameterized for drug-like small molecules and their interactions with proteins, making it an excellent choice for applications like flexible docking, which involves molecular dynamics in a docking simulation.

#### 3.3.3. Docking Validation

For molecular docking (Maestro Version 11.1.012, Release 2017-1), the Glide algorithm (Release 2017-1; Schrödinger LLC, New York, NY, USA) was applied in a hierarchical workflow, followed by Induced Fit Docking (IFD) with Prime side-chain refinement. Approximately 20 poses per ligand were generated at each docking stage, and the top-ranking poses were advanced to the next level of accuracy. Population size and sampling parameters were those internally defined by the Glide protocol. No crystallographic water molecules were retained in the docking grids to focus on direct ligand–protein interactions.

Receptor grids were generated by centering the grid boxes on the co-crystallized ligands **axitinib** (for VEGFR-2, PDB ID: 4AG8 [7]) and **ARS-1620** (for K-RAS, PDB ID: 5V9U [8]). The enclosing grid box was defined as a cube with side lengths of 26 Å and a grid point spacing of 2.89 Å, ensuring complete coverage of the active sites while allowing adequate space for ligand flexibility and induced-fit adjustments.

The receptor grids were centered on the co-crystallized ligands **axitinib** (VEGFR-2, PDB ID: 4AG8) and **ARS-1620** (K-RAS, PDB ID: 5V9U), with grid center coordinates X = 20.82, Y = 25.54, Z = 39.46 Å for VEGFR-2 and X = −8.42, Y = 8.02, Z = 13.27 Å for K-RAS, ensuring accurate representation of the functional binding sites.

The Virtual Screening Workflow (VSW) was employed, comprising a three-tiered docking protocol of increasing precision. Initially, High-Throughput Virtual Screening (HTVS) was performed, retaining 50% of the top-scoring ligands, which proceeded to Standard Precision (SP) docking. Subsequently, 50% of the ligands from the SP step advanced to the final stage, which involved Extra Precision (XP) docking. Employing the Extra Precision (XP) mode as the scoring function, 3D conformers were docked into the receptor model. Notably, the docking protocol successfully redocked the original ligands within the receptor-binding pockets with an RMSD < 0.51 Å. The Induced Fit Docking (IFD) simulation was conducted using the Schrödinger IFD protocol, a highly accurate and reliable method that accounts for the flexibility of both ligand and receptor [12,13]. The resulting IFD poses were ranked based on the IFD score, calculated as: IFD score = 1.0 × Glide Gscore + 0.05 × Prime Energy, which integrates both protein–ligand interaction energy and the overall system energy.

#### 3.3.4. Molecular Dynamics Simulation

Molecular Dynamics (MD) simulations were carried out using the Desmond software (Maestro Version 13.8.135, Release 2023-4, Schrödinger LLC, New York, NY, USA) to evaluate the stability and binding affinity of compounds **623292**, **625894**, **737734**, and **753203**, as well as their respective reference drugs previously discussed, in complex with K-RAS and VEGFR-2. The simulations were conducted under the constant temperature and pressure ensemble (NPT), ensuring accurate regulation of thermodynamic conditions. Pressure equilibration within the NPT ensemble was achieved through volume adjustments, allowing the unit cell vectors to vary accordingly. Simulation parameters were configured with a system temperature of 300 K and a pressure of 1013.25 bar. The Nose-Hoover Chain thermostat and the Martyna-Tobias-Klein barostat were settled with a relaxation time of 1 ps and 2 ps, respectively. Before commencing the production run, the systems underwent energy minimization for 1000 steps to establish a stable starting point. Subsequently, a production run of 50 ns was conducted for compounds **623292**, **625894**, **737734**, **753203**, **axitinib**, **cediranib**, **semaxanib**, **tivozanib** in complex with VEGFR-2, and for compounds **623292**, **625894**, **737734**, **753203**, **adagrasib**, **garsorasib**, **glecirasib**, **sotorasib**, and **ARS-1620** in complex with K-RAS. Next, a longer simulation of 200 ns was performed for compounds **737734**, **753203**, **axitinib**, **cediranib**, **semaxanib**, and **tivozanib** in complex with VEGFR-2, and for compounds **737734**, **753203**, **adagrasib**, and **ARS-1620** in complex with K-RAS. The simulation results were meticulously analyzed, enabling the extraction of key parameters such as Root Mean Square Deviation (RMSD), Ligand Root Mean Square Fluctuation (L-RMSF), Protein–Ligand contacts, Radius of Gyration (rGyr), Intramolecular Hydrogen Bonds (intraHB), Molecular Surface Area (MolSA), Solvent Accessible Surface Area (SASA), and Polar Surface Area (PSA). This comprehensive evaluation provides fundamental insights into the dynamic behavior of the complexes, offering valuable information regarding their structural stability and the nature of molecular interactions throughout the simulation period.

#### 3.3.5. MM-GBSA Analysis

The Molecular Mechanics with Generalized Born and Surface Area (MM-GBSA, Release 2017-1; Schrödinger LLC, New York, NY, USA) calculations were employed to estimate the binding free energies of protein–ligand interactions, encompassing components such as van der Waals, electrostatic, polar solvation, hydrogen bonding, and overall binding energies. In this study, the MM-GBSA approach was applied using a single-step calculation method, adopting the VSGB solvation model and the OPLS-2005 force field.

## 4. Conclusions

According to the pivotal role of angiogenesis in supplying nutrients and oxygen to tumor cells through the formation of new blood vessels from pre-existing vasculature, this process has been established as a key target in cancer therapy. In details, VEGFR-2 and K-RAS were selected as promising therapeutic targets. Their potential to disrupt angiogenesis is due to their involvement in the dysregulation of the signaling pathways, critical for the survival and proliferation of endothelial cells, and consequently of malignant cells, making them favorable targets.

In this study, we employed a hybrid computational approach aimed at identifying novel potential inhibitors capable of targeting key proteins involved in the angiogenesis pathway. Our protocol included a meticulous cleaning phase of the NCI database, using QikProp and SwissADME tools to predict the pharmacokinetic profiles of the compounds and eliminate those lacking drug-like characteristics. The refined database was then screened using the Biotarget Predictor Tool (BPT) in multitarget mode to identify molecules with simultaneous affinity for both VEGFR-2 and K-RAS. The 780 top-scoring structures were subsequently subjected to a hierarchical structure-based analysis, comprising HTVS, SP, XP, and IFD, enabling the identification of the four best-ranked ligands for further investigation. Molecular Dynamics Simulations confirmed the high stability of compound **737734** in complex with both VEGFR-2 and K-RAS, supporting its potential as a lead multitarget inhibitor.

While these findings are promising, it is important to acknowledge the limitations of in silico approaches. The predictive accuracy of virtual screening tools, docking scores, and force-field–based simulations is inherently approximate, and false positives or negatives may occur. However, combining diverse techniques is essential to address the challenges associated with in silico evaluations, allowing to reduce the risk of false positives or negatives. Therefore, the results reported here should be regarded as a prioritization strategy to accelerate drug discovery, rather than definitive proof of biological activity.

Overall, this study demonstrates the feasibility of combining ligand-based and structure-based computational approaches for the rational identification of multitarget antiangiogenic agents. The multitarget activity allows for improved patient compliance, reduced risk of adverse drug interactions, and a more straightforward identification of both the desired therapeutic effects and potential undesired reactions.

The proposed workflow allowed us to identify and prioritize novel dual inhibitors with potential therapeutic relevance. Future work will focus on experimental validation, including biochemical binding and enzymatic inhibition assays, followed by in vitro cell-based studies to confirm their anticancer and antiangiogenic properties. In a broader perspective, this approach may accelerate the rational development of multitarget anticancer therapies and contribute to more effective strategies in drug discovery.

## Figures and Tables

**Table 1:** IFD scores of the top 23 ranked ligands, alongside the reference co-crystallized ligands ARS-1620 and **axitinib** against K-RAS (PDB code 5V9U) and VEGFR-2 (PDB code 4AG8) [24,28], as well as the well-known inhibitors **adagrasib**, **garsorasib**, **glecirasib**, and **sotorasib** for K-RAS, and **cediranib**, **semaxanib**, and **tivozanib** for VEGFR-2.

K-RAS		VEGFR-2	
Title	IFDScore	Title	IFDScore
**ARS-1620**	−466.025	**753203**	−669.154
**adagrasib**	−465.595	**737734**	−668.884
**753203**	−464.212	**cediranib**	−667.339
**625894**	−462.446	**axitinib**	−667.088
**737734**	−462.348	**tivozanib**	−666.343
**623292**	−462.176	**623292**	−664.999
**654632**	−460.126	**641220**	−664.389
**751605**	−459.988	**625894**	−663.527
**766494**	−459.022	**720567**	−663.426
**736273**	−458.777	**697231**	−663.010
**697231**	−458.666	**751605**	−662.414
**735330**	−458.103	**678359**	−662.108
**169874**	−457.836	**735330**	−661.753
**641220**	−457.630	**707444**	−660.894
**697673**	−457.491	**747856**	−660.605
**747856**	−457.411	**697673**	−660.516
**744650**	−456.930	**744650**	−660.441
**707444**	−456.861	**766494**	−660.166
**707776**	−456.732	**169874**	−660.023
**720567**	−456.708	**736273**	−659.949
**733308**	−456.185	**697227**	−659.573
**647945**	−456.120	**654632**	−659.458
**sotorasib**	−455.351	**733308**	−659.224
**665325**	−455.236	**665325**	−658.868
**garsorasib**	−455.067	**707776**	−656.454
**697227**	−454.700	**647945**	−656.049
**glecirasib**	−453.644	**semaxanib**	−655.604
**678359**	−452.451		

**Table 2:** Overview of the amino acids involved in the binding of the selected compound **737734** in the binding sites of K-RAS and VEGFR-2, compared to co-crystalized ligands **ARS-1620** and **axitinib**, at 4 Å proximity.

K-RAS			VEGFR-2		
Title	ARS-1620	737734	Title	Axitinib	737734
Val^7^		X	Lys^838^	X	
Val^9^	X	X	Leu^840^	X	X
Gly^10^	X	X	Val^848^	X	X
Ala^11^	X	X	Ala^866^	X	X
Cys^12^	X	X	Val^867^	X	
Lys^16^	X	X	Lys^868^	X	X
Pro^34^	X		Glu^885^	X	
Thr^35^	X		Ile^888^		X
Thr^58^	X	X	Leu^889^	X	X
Ala^59^	X	X	Ile^892^		X
Gly^60^	X		Val^898^		X
Gln^61^	X	X	Val^899^	X	X
Glu^62^	X	X	Leu^901^		X
Glu^63^		X	Val^914^	X	X
Tyr^64^	X	X	Ile^915^	X	X
Arg^68^	X	X	Val^916^	X	X
Asp^69^	X		Glu^917^	X *	
Tyr^71^	X	X	Phe^918^	X	X
Met^72^	X	X	Cys^919^	X *	X
Hie^95^	X *	X *	Lys^920^	X	X
Tyr^96^	X	X *	Phe^921^	X	X
Gln^99^	X	X	Gly^922^	X	X
Ile^100^	X	X	Leu^1019^		X
Arg^102^	X	X	Leu^1035^	X	X
Val^103^	X		Ile^1044^		X
			Cys^1045^	X	X
			Asp^1046^	X *	X *
			Phe^1047^	X	X
Tot.	23	20	Tot.	22	25

* H-bonds.

**Table 3:** Summary of MM-GBSA analysis: comparative binding energies of reference ligands ***ARS-1620*** and ***axitinib***, and compound **737734** in complex, respectively, with K-RAS and VEGFR-2.

**K-RAS**					
**Complex**	**Prime Coulomb**	**Prime vdW**	**Prime Solvent**	**Prime Hbond**	**Prime Energy**
**737734**	−6302.17	−851.78	−1609.49	−133.84	−9040.7
**ARS-1620**	−6305.19	−847.79	−1626.10	−133.56	−9063.7
**VEGFR-2**					
**Complex**	**Prime Coulomb**	**Prime vdW**	**Prime Solvent**	**Prime Hbond**	**Prime Energy**
**737734**	−9172.15	−1549.90	−1824.98	−142.24	−13,112.3
**Axitinib**	−9151.63	−1578.12	−1744.62	−145.96	−13,056.9

## Data Availability

The original contributions presented in this study are included in the article/Supplementary Material. Further inquiries can be directed to the corresponding author.

## Supplementary Materials

The following supporting information can be downloaded at https://www.mdpi.com/article/10.3390/ph18101579/s1, Table S1: DAS values of NCI structures for each target (K-RAS and VEGFR-2); Table S2: Multitarget Score for NCI compounds; Table S3: top 100 small molecules from each XP docking simulation; Figure S1: Summary of the tracked protein Secondary Structure Elements (SSE) for K-RAS; Figure S2: Summary of the tracked protein Secondary Structure Elements (SSE) for VEGFR-2; Figure S3: In-depth structural analyses for complex 737734/K-RAS, in details, calculated rGyr, intraHB, MolSA variations, SASA changes, and PSA dynamics over 200 ns simulation; Figure S4: In-depth structural analyses for complex 737734/VEGFR-2, in details, calculated rGyr, intraHB, MolSA variations, SASA changes, and PSA dynamics over 200 ns simulation; Figure S5: Calculated P-RMSF during the simulation for K-RAS and VEGFR-2.
