# Supplementary material for: A Hybrid In Silico Approach for Identifying Dual VEGFR/RAS Inhibitors as Potential Anticancer and Anti-Angiogenic Agents
Source: Pharmaceuticals (Basel). 2025 Oct 18;18(10):1579. doi: 10.3390/ph18101579 (PMC12566778; doi:10.3390/ph18101579)
Supplement: Supplementary file 1 [file pharmaceuticals-18-01579-s001.zip › Supplementary Material-Figures.pdf]

Figures S1–S2 summarize the analysis of Secondary Structure Elements (SSE) for K-RAS and VEGFR-2. Specifically, Figures S1a and S2a display the distribution of SSE along the residue index across the full length of the protein structures. The corresponding Figures S1b and S2b present a frame-by-frame overview of SSE composition throughout the trajectory, offering a dynamic representation of structural variations during the simulation period. Finally, the bottom panels (Figures S1c and S2c) track the SSE assignments for each residue over time, delivering a detailed and time-resolved view of secondary structure evolution. Collectively, these plots provide a robust and multifaceted depiction of SSE dynamics, supporting a deeper understanding of protein conformational behavior during molecular dynamics simulations.

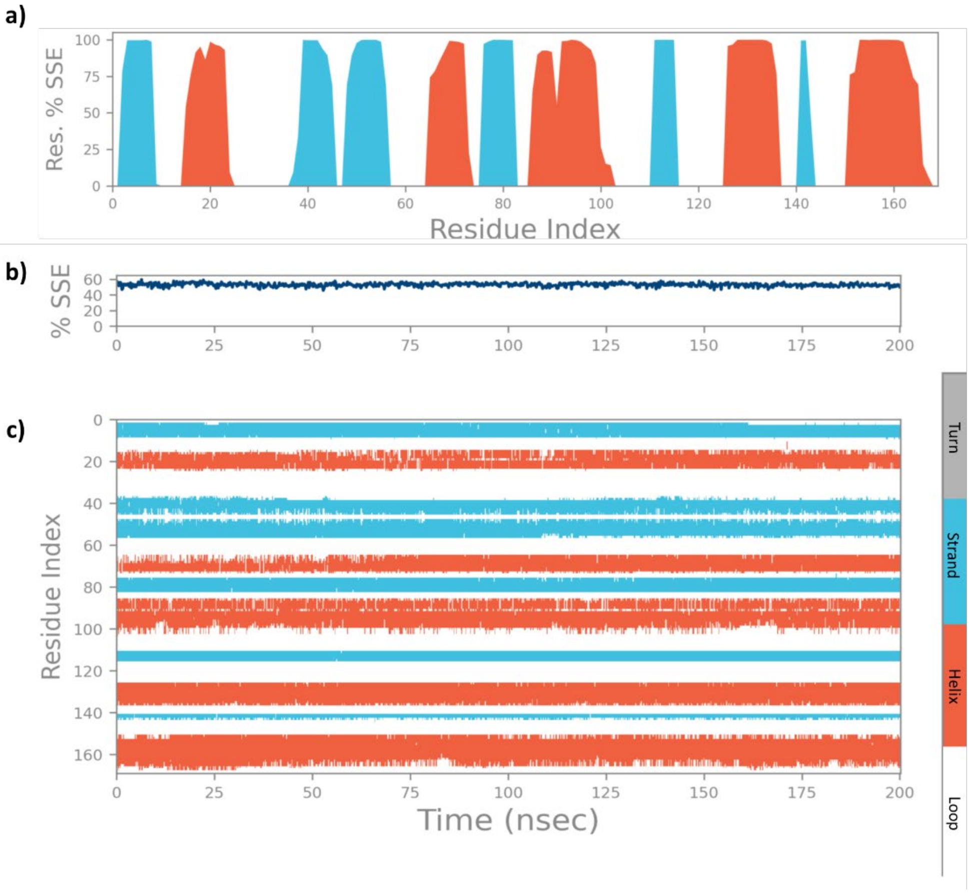

Figure S1. K-RAS protein secondary structure elements (SSE) evaluation: **(a)** distribution of SSE by residue index across the entirety of the protein structure; **(b)** dynamic snapshot of structural changes over the simulation duration; **(c)** SSE assignment for each residue over time.

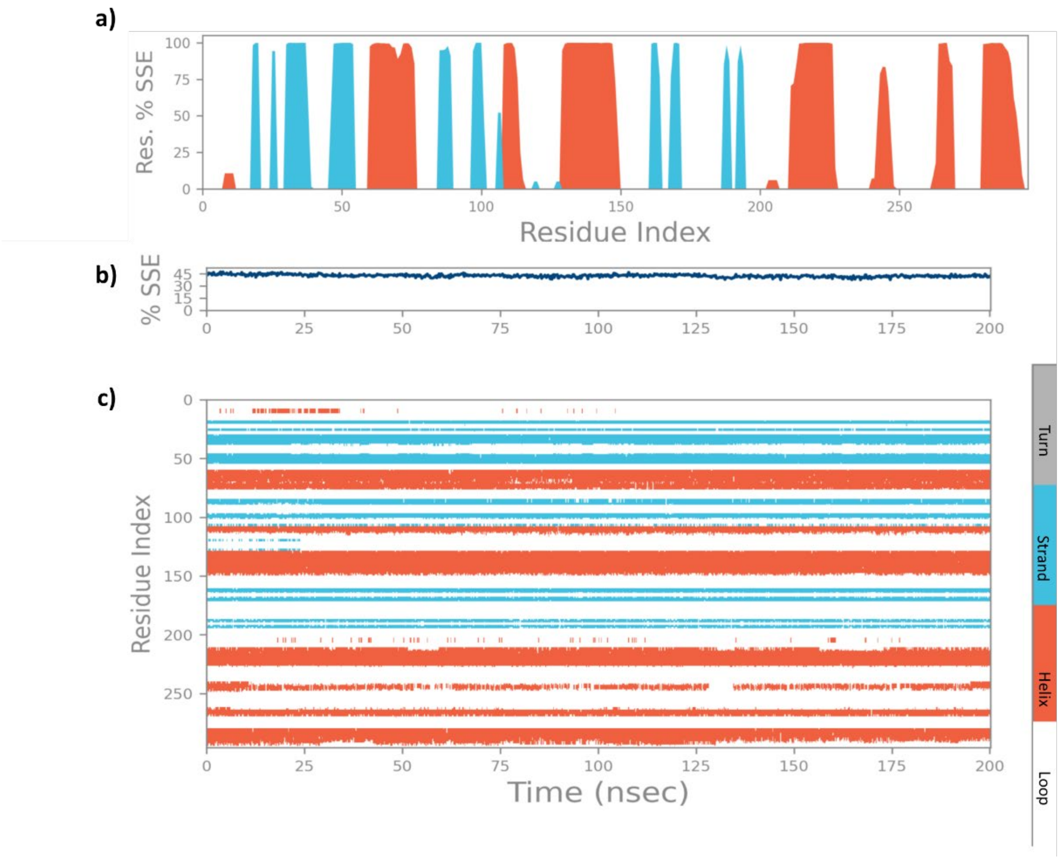

Figure S2. VEGFR-2 protein secondary structure elements (SSE) evaluation: **(a)** distribution of SSE by residue index across the entirety of the protein structure; **(b)** dynamic snapshot of structural changes over the simulation duration; **(c)** SSE assignment for each residue over time.

In-depth structural analyses, based on multiple parameters, were performed for each complex to elucidate key molecular features. The Radius of Gyration (rGyr) was first calculated to assess the degree of "extendedness" of the ligand, by measuring the RMS distance of each atom from the ligand's center of mass—an indicator of its overall compactness or spatial dispersion.

Concurrently, the number of Intramolecular Hydrogen Bonds (intraHB) was determined, reflecting the internal hydrogen-bonding network within the ligand. The Molecular Surface Area (MolSA) was also computed, quantifying the total surface area of the ligand in each complex.

To evaluate solvent interaction, the Solvent Accessible Surface Area (SASA) was calculated, highlighting the ligand surface exposed to solvent molecules. In parallel, the Polar Surface Area (PSA) was assessed to quantify the surface area occupied by polar atoms.

The comprehensive results of these analyses, reported in Figures S3 and S4 for the **737734**/K-RAS and **737734**/VEGFR-2 complexes, respectively, offer a detailed and comparative view of the ligand's structural behavior across different biological environments.

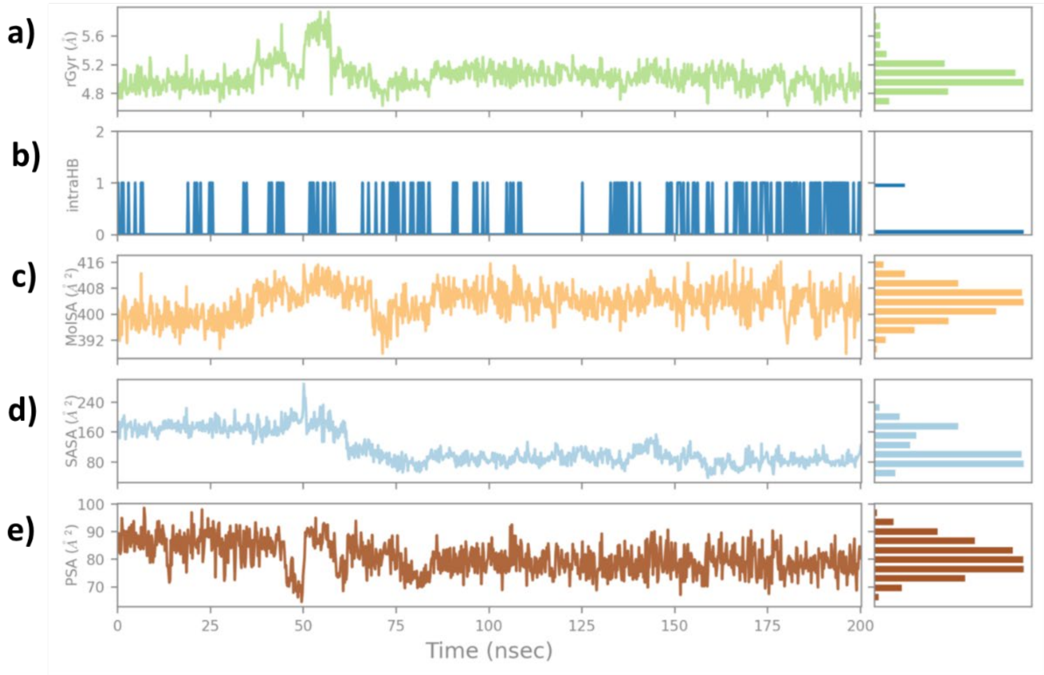

Figure S3. In-depth structural analyses for complex **737734**/K-RAS: (a) calculated rGyr over a 200ns simulation; (b) intraHB over 200ns; (c) MolSA variations over the 200ns simulation; (d) SASA changes during the 200ns simulation; (e) PSA dynamics over 200ns simulation.

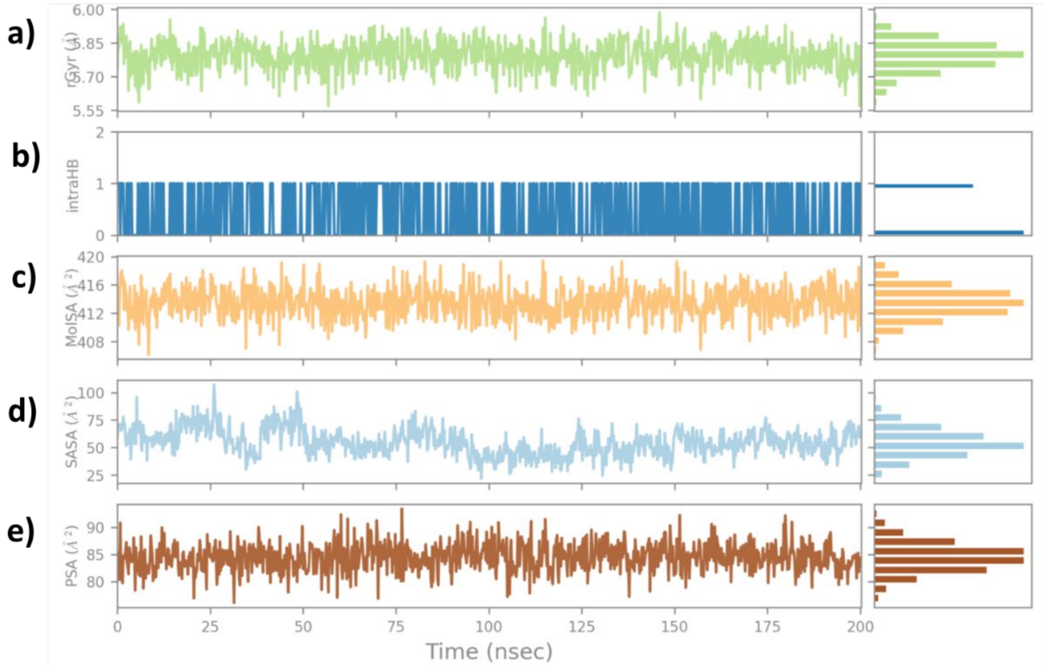

Figure S4. In-depth structural analyses for complex **737734**/VEGFR-2: **(a)** calculated rGyr over a 200ns simulation; **(b)** intraHB over 200ns; (c) MolSA variations over the 200ns simulation; (**d**) SASA changes during the 200ns simulation; **(e)** PSA dynamics over 200ns simulation.

Finally, Figure S5 presents the Protein Root Mean Square Fluctuation (P-RMSF) plots, with graphs S5a and S5b corresponding to K-RAS and VEGFR-2, respectively. The RMSF quantifies structural fluctuations as a function of residue or atom, providing a measure of local flexibility. Specifically, in this analysis, fluctuations are evaluated based on the alpha carbon (Cα) atoms, offering residue-level insight into protein dynamics.

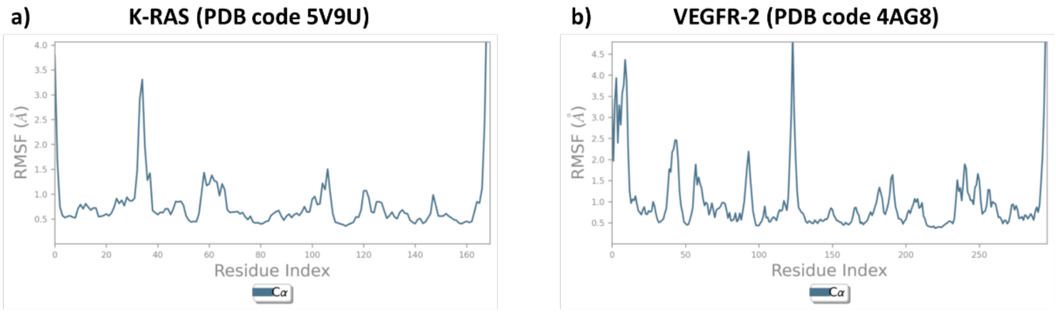

Figure S5. (a) Calculated P-RMSF during the simulation for K-RAS; **(b)** calculated P-RMSF during the simulation for VEGFR-2.
